# Association of a Bacteriophage with Meningococcal Disease in Young Adults

**DOI:** 10.1371/journal.pone.0003885

**Published:** 2008-12-09

**Authors:** Emmanuelle Bille, Roisin Ure, Stephen J. Gray, Edward B. Kaczmarski, Noel D. McCarthy, Xavier Nassif, Martin C. J. Maiden, Colin R. Tinsley

**Affiliations:** 1 Institut National de la Santé et de la Recherche Médicale, Paris, France; 2 Unité de pathogénie des infections systémiques, Université Paris Descartes, Faculté de Médecine, Paris, France; 3 Laboratoire de Microbiologie, Assistance Publique-Hôpitaux de Paris, Hôpital Necker-Enfants Malades, Paris, France; 4 Department of Zoology, Peter Medawar Building for Pathogen research, Oxford, United Kingdom; 5 Meningococcal Reference Unit, Health Protection Agency, Manchester Royal Infirmary, Manchester, United Kingdom; 6 Laboratoire de Microbiologie et Génétique Moléculaire, UMR AgroParisTech/INRA 1238/CNRS 2585, AgroParisTech, Centre de Grignon, Thiverval-Grignon, France; University of Florida, United States of America

## Abstract

Despite being the agent of life-threatening meningitis, *Neisseria meningitidis* is usually carried asymptomatically in the nasopharynx of humans and only occasionally causes disease. The genetic bases for virulence have not been entirely elucidated and the search for new virulence factors in this species is hampered by the lack of an animal model representative of the human disease. As an alternative strategy we employ a molecular epidemiological approach to establish a statistical association of a candidate virulence gene with disease in the human population. We examine the distribution of a previously-identified genetic element, a temperate bacteriophage, in 1288 meningococci isolated from cases of disease and asymptomatic carriage. The phage was over-represented in disease isolates from young adults indicating that it may contribute to invasive disease in this age group. Further statistical analysis indicated that between 20% and 45% of the pathogenic potential of the five most common disease-causing meningococcal groups was linked to the presence of the phage. In the absence of an animal model of human disease, this molecular epidemiological approach permitted the estimation of the influence of the candidate virulence factor. Such an approach is particularly valuable in the investigation of exclusively human diseases.

## Introduction

Despite its notoriety as the causative agent of meningococcal disease, which kills an estimated 50,000 individuals worldwide annually [1], *Neisseria meningitidis* is a frequent asymptomatic coloniser of the human nasopharynx. Only a very small proportion of infections proceed to a sustained bacteraemia and thence to meningitis or septicaemia. Analysis of results from multilocus enzyme electrophoresis (MLEE [2]) and multilocus sequence typing (MLST [3]) have demonstrated the existence of distinct phylogenetic groupings (termed lineages, or clonal complexes), a small number of which are consistently more likely to be isolated from patients than are others [4]. These are the so-called hyper-virulent, or hyperinvasive, clonal complexes [3], which are responsible for the large majority of meningococcal disease worldwide.

The reasons for which disease occurs in some individuals and not in others remain unclear, but both human and bacterial factors are likely to be important in determining the outcome of infection. The most important factors predisposing individuals to invasive disease are the absence of bactericidal antibodies [5], [6] and dysfunction in the complement system [7]. The importance of bacterial factors is exemplified by the absolute necessity of the protective polysaccharide capsule for survival in the bloodstream and subsequent disease. Several other bacterial structures have been shown by laboratory assays to be important in pathogenesis, such as type IV pili [8] and iron acquisition systems [9]. There are no known virulence factors that are specific to hyperinvasive clonal complexes. For example, many meningococci belonging to clonal complexes that are not hyperinvasive are encapsulated and have iron acquisition sytems; pili are required for colonisation of the human nasopharynx whether or not this is followed by disease. Furthermore, some established or potential virulence factors are not universally present in meningococci isolated from invasive disease. The pathogenicity of *Neisseria meningitidis* is multifactorial and it is likely that many determinants important for the invasive phenotype remain to be discovered.

Typical strategies for the discovery of novel virulence factors in pathogens include the screening of banks of mutants, the mutation of candidate genes chosen on the basis of homology to known virulence factors and whole genome comparisons, often using DNA arrays to detect genes specific to pathogenic strains. Either as an integral part of these techniques or as a subsequent validation step, the effect of deletions of the identified gene will be evaluated in animal or other laboratory models. However, for many human diseases, and in particular in the case of meningococcal meningitis, the difficulty of reproducing the human disease in animal models has hampered the identification of novel virulence factors. An alternative to such methods consists in molecular epidemiological studies in the community, involving the analysis of isolates from human disease and asymptomatic carriage. These studies are founded on the expectation that the presence of a virulence gene is correlated with a higher probability of being isolated from invasive disease.

In a previous study [10] we compared the genomes of meningococci belonging to clonal complexes designated as having higher or lower invasive potential [11], [12] and identified an 8-kb genetic island which was significantly associated with meningococci causing invasive disease and which was named MDA for Meningococcal Disease-Associated island. Subsequent analyses suggest that the element corresponded to an integrated bacteriophage. Here, the presence of the MDA phage is surveyed in a collection of 1288 meningococci, isolated from both disease and carriage in south-east England between 1999 and 2001. The data demonstrate that a large part of the invasiveness of strains belonging to hyperinvasive clonal complexes is correlated with the presence of the phage, and show an association with virulence in young adults but not in children less than two years of age.

## Methods

### Meningococcal isolates

A collection of 1288 meningococcal isolates, collected in South East England in 1999 and 2001, was surveyed for the presence of the MDA phage. A total of 703 of these isolates were collected before the introduction of the meningococcal conjugate C polysaccharide vaccine and consisted of 500 meningococci isolated from a carriage study performed in the Oxford area between November and December 1999 [13] and 203 clinical isolates collected from cases in south-east England between November 1998 and October 1999 and submitted to the England and Wales National Reference Unit at Manchester (MRU). The remaining 585 isolates were collected after the vaccine introduction and comprised 431 isolates from carriage, isolated in Oxford between October 2001 and December 2001, and 154 meningococci isolated from cases of invasive disease in south-eastern England and submitted to the MRU between November 2000 and October 2001. Healthy carriers gave written consent for genetic characterisation of meningococci isolated from their throats as described [13]. The disease isolates were obtained from patients on admission to hospital as part of the standard procedure for diagnosis and treatment of meningococcal disease. Further information concerning the isolates is provided in Table S1.

### DNA techniques, PCR methods and oligonucleotide primers

Meningococci were grown on Columbia blood agar plates (Oxoid; containing 5% defibrinated horse blood) and chromosomal DNA prepared with the Wizard Genomic DNA Purification kit (Promega). The presence or absence of the MDA phage was determined by long-range PCR amplification as described [10]. The possession of one or more copies of the MDA was detected by the production of an amplification product of about 8 kbp. In order to diminish problems caused by potential inhibitors of the PCR present in the DNA preparations, when the initial result (using about 0.5 µg of chromosomal DNA) was negative, the reaction was repeated using a 1/10 dilution of the DNA preparation.

To confirm the amplification data a dot blot reaction was performed. The DNA preparations (1 µg) were spotted onto nylon membranes and reacted with probes prepared by PCR, corresponding to approximately 700 bp fragments of ORFs 1, 6 and 9 of the MDA phage of strain Z2491 [10], and labelled with ^32^P. The oligonucleotides used were as follows: for ORF1; GGCATAACGCGCAAATGCAAATCA and CAGGTTCTAGCCCTTTGGGA (producing a fragment of length 757 bp), for ORF6; GGGAAAAGTTAAGGAATCGTCCTGA and CAGACGGCAGATTTAAATCTTCTGC (producing a fragment of 726 bp) and for ORF9; GGCATGGAGGCAACAGGCATCTATT and GGCTTACCCGCTTTTTTCAGATTAT (producing a fragment of 689 bp). Isolates giving discordant results for the amplification and dot-blot experiments were retested.

### Statistical analysis

The association of disease with both the presence of the MDA phage and population structure as indexed by clonal complex was measured as odds ratios using a logistic regression model. The association of each of the larger clonal complexes with disease was modelled using a categorical clonal complex variable for the larger clonal complexes (those containing 15 or more isolates, being the ST-8, 11, 22, 23, 35, 41/44, 53, 60, 103, 167, 198, 213, 269 and 1157 clonal complexes) and combining isolates not assigned to clonal complexes and those from clonal complexes represented by 14 or fewer isolates in the dataset into a single category. An unadjusted model was undertaken for the full dataset for each of MDA and clonal complex. Multivariable models were used to consider the independent effects of clonal complex and the presence of the MDA prophage. The MDA prophage–disease association was modelled with clonal complex included as a categorical variable. Separate models were run for each major clonal complex comparing isolates within that complex with the rest of the dataset and adjusting for the presence of MDA. In the adjusted logistic regression model isolates from clonal complexes with outcome predicted completely by the model (ST-53 and ST-198 complexes, present only in the carriage dataset) are dropped by the model. These isolates are also excluded from the unadjusted model to allow comparison between the two models. This results in a reduced dataset (n = 1186 for all ages, n = 1105 when considering disease in children only and n = 790 for disease in those aged 11–30).

The distribution of MDA phage among disease-causing isolates by age group was tested using a Chi square test. For the purposes of this analysis, the isolates were divided into four groups of equal size on the basis of patient age (less than two years old, two to 12, 13 to 28 and 29 or older). Two of these groups (the age brackets 0 to 1 years and 13 to 28 years) correspond to periods of peak incidence of disease in young children and in young adulthood.

## Results

### Association of pathogenicity both with membership of hyperinvasive clonal complexes and with possession of the MDA phage

The MDA phage was present in 48% of the carried isolates at both sampling times (240/500 isolates in 1999 and 205/431 isolates in 2001). It was present in 82.3% (167/203) of disease isolates in 1999 and in 88.4% (136/154) of disease isolates in 2001. The prevalence of the MDA phage in different clonal complexes varied from 98% in the ST-11 complex, to none in the ST-53 clonal complex (Tables 1 and S2).

**Table 1:** Distribution of the MDA Phage among Clonal Complexes.

Clonal complex	Number of isolates			Proportion with MDA phage (%)
	Without MDA phage	With MDA phage	Total	
ST-11 clonal complex	2	107	109	98.2
ST-32 clonal complex	1	29	30	96.7
ST-41/44 clonal complex	9	197	206	95.6
ST-269 clonal complex	6	112	118	94.9
ST-35 clonal complex	3	32	35	91.4
ST-1157 clonal complex	6	59	65	90.8
ST-8 clonal complex	2	13	15	86.7
ST-23 clonal complex	21	33	54	61.1
ST-103 clonal complex	15	6	21	28.6
ST-167 clonal complex	31	9	40	22.5
ST-60 clonal complex	76	10	86	11.6
ST-213 clonal complex	51	5	56	8.9
ST-22 clonal complex	108	10	118	8.5
ST-198 clonal complex	27	2	29	6.9
ST-53 clonal complex	73	0	73	0
Unassigned	86	95	181	52.5

The major disease-causing serogroups were B and C (responsible respectively for 60 and 31% of disease). Non-groupable organisms caused 3% of disease while they constituted 55% of the carrier isolates. The phage was harboured by 78 percentage of group B, 96% of group C and 44% of non-groupable isolates, these figures differing by less than 7% between pre-and post-vaccination samples.

Meningococcal isolates containing the MDA phage were more likely to be isolated from invasive disease than from carriage in the whole data set (OR = 4.9; 95% CI 3.6–6.7) with no significant difference in the pre- and post-vaccination samples (1999: OR = 3.9, CI 2.6–5.8; and 2001: OR = 6.7, CI 3.9–11.4). Clonal complex was also strongly associated with disease in the unadjusted analysis (Table 2, unadjusted analysis), in agreement with the studies which form the basis for the definition of hyperinvasive complexes [3], [4]. Consequently, after adjustment for the existence of the different clonal complexes, the odds ratio for association between MDA and disease was reduced (1.1; 95% CI 0.6–1.8) again showing no significant difference between the pre- and post-vaccine samples (1999: OR = 0.6, 95% CI 0.3–1.3; 2001: OR 1.8, 95% CI 0.8–3.9).

**Table 2:** Estimated Contribution of MDA to the Invasive Phenotype of Meningococci.

Hyper-invasive lineage	Number of isolates		Odds ratio of disease association [95% CI]		Estimated proportion of invasiveness attributable to MDA
	Carriage	Disease	Unadjusted	Adjusted for MDA presence	
ST-11 complex	11	98	31.6 [16.7–59.9]	19.6 [10.3–37.5]	0.38
ST-8 complex	3	12	10.8 [3.0–38.4]	8.5 [2.2–32.5]	0.21
ST-32 complex	12	18	4.0 [1.9–8.5]	2.4 [1.1–5.1]	0.40
ST-269 complex	58	60	3.0 [2.1–4.5]	1.8 [1.2–2.7]	0.40
ST-41/44 complex	108	98	2.9 [2.1–3.9]	1.6 [1.2–2.2]	0.45

Hence, in order to take into account the confounding effect of the meningococcal population structure (that is, to distinguish the effects of clonal complex and of possession of the MDA phage on virulence), we examined the effect of MDA on the disease-causing potential of each of the 5 major hyperinvasive clonal complexes separately, relative to the other complexes. Significantly, adjustment for the presence of MDA diminished the apparent association of disease with clonal complex by between 21% and 45% (Table 2), implying that 21 to 45% of the invasive potential of these clonal complexes is associated with the presence of the MDA.

### The association of the MDA phage with disease in young adults

**Figure 1:**
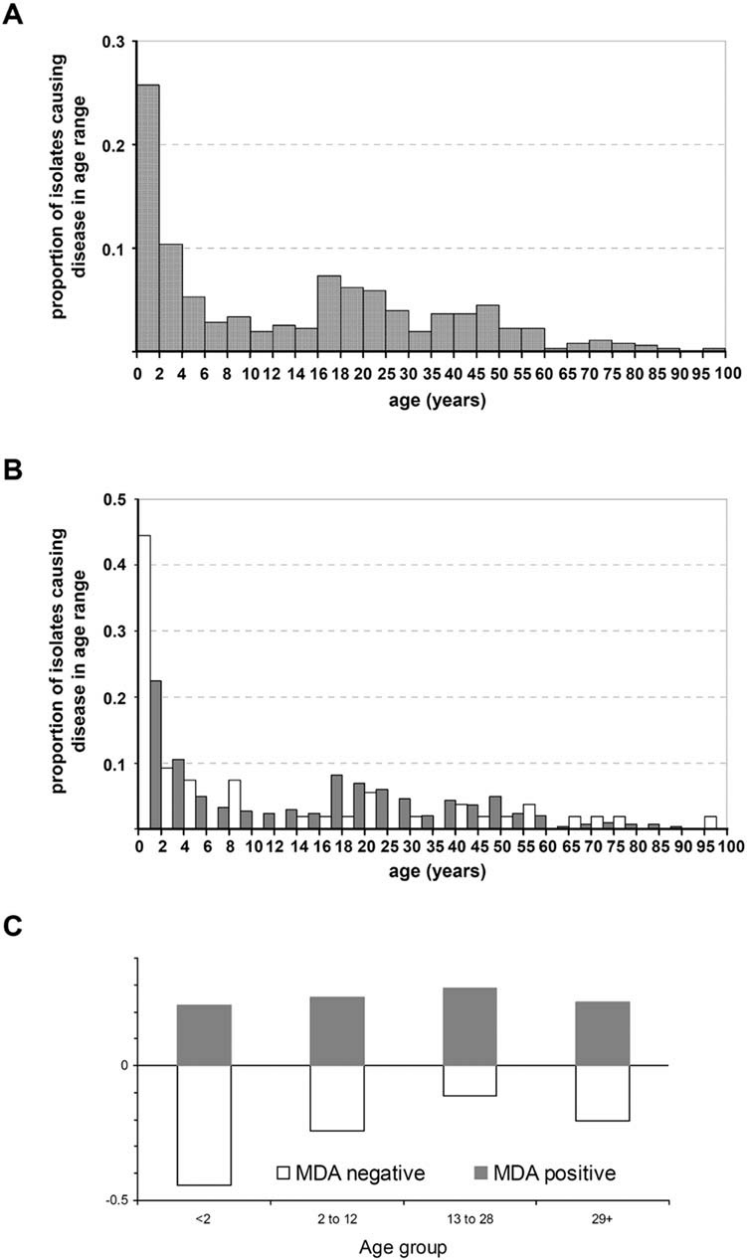
Distribution of the integrated phage in disease isolates by age of patient. (A) Comparison of the age distribution of meningococcal disease in the population studied. (B) Comparison of the age distribution of disease caused by MDA-positive (grey bars) and by MDA-negative (white bars) meningococci. Numbers of cases in each of the two groups are separately normalised to unity. (C) Relative proportions of disease in each of four age groups caused by MDA-positive or MDA-negative meningococci. The age groups were chosen in order to contain approximately equal numbers of meningococcal isolates. Two of these (the age bracket 0 to 2 years and 13 to 28 years) correspond to the periods of peak incidence of disease, respectively in infancy and in young adulthood.

The age distribution of the patients (Fig. 1A) was typical of meningococcal disease, with a peak incidence in children under the age of two years and a smaller peak of incidence in young adults [6], [14]. The age distribution of disease due to MDA-containing meningococci, appeared different to that of disease caused by MDA-negative bacteria (Fig. 1B), a difference that was shown to be statistically significant (p = 0.003, global Chi^2^ for non-random distribution; Fig. 1C). The results indicated a negative association of the phage with children under two years of age, a strong positive association of the phage with disease isolates in young adults aged 13 to 28 years, the age group which forms the main reservoir for *N. meningitidis*, and an intermediate distribution in the other age groups (2 to 13 years, and over 29 years).

A separate analysis of the association of MDA with disease, divided into 2 age groups with those with disease aged 0–10 years and those aged 11–30 considered separately (Table 3), showed no evidence for association of MDA with disease, independently of clonal complex, in the 0–10 age group, but a substantial association among adolescents and young adults (adjusted odds ratio 3.9; 95% CI 1.1–13.6).

**Table 3:** Odds Ratio for Association between MDA and Disease with and without Adjustment for Clonal Complex Restricted by Age Group.

	Age 0–10*	Age 11–30*
Model	(n = 1005)	(n = 790)
Unadjusted OR	3.3 [2.2–4.8]	13.8 [6.0–31.9]
Adjusted† OR	0.7 [0.4–1.3]	3.9 [1.1–13.6]

* Cases are divided into the age groups 0–10 years and 11–30 years. Carried strains are carriage isolates from adolescents and young adults. Sample sizes exclude isolates dropped from the models due to complete prediction as indicated in the methods.

† Adjustment is for clonal complex modelled as a categorical variable. Clonal complexes with less than 15 isolates and unassigned STs are grouped together. Clonal complexes with 15 or more isolates are 8, 11, 22, 23, 32, 35, 41/44, 53, 60, 103, 167, 198, 213, 269, 1157.

## Discussion

The capsular polysaccharide remains the principal virulence determinant for meningococci, with virtually all invasive disease isolates elaborating a polysaccharide capsule corresponding to one of the disease-associated serogroups (A, B, C, Y, W-135 and X). In addition, iron acquisition systems and type IV pili are probably also necessary for pathogenesis, but while possession of each of these factors is necessary for invasive disease it is not sufficient. It is likely that many other genetic factors, which remain largely undefined, contribute to the capacity of a given meningococcus to cause invasive disease.

Genomic comparisons using microarrays permit the detection of candidate pathogenicity genes on the basis of their consistent presence in invasive and their absence from less virulent organisms. Typical methods of affirming the practical importance of putative virulence factors require that the inactivation of a gene have a measurable effect in an animal or *ex vivo* model of pathogenesis. However, in the case of the meningococcus, an exclusively human pathogen, such models are not representative of the disease process and a factor promoting virulence in humans may not be detected. The analysis of isolate collections from carriage and disease in the community permits the confirmation of potential virulence factors on the basis of a statistical association with human disease, and circumvents the limitations of laboratory models. We hypothesised firstly that a virulence gene would be overrepresented in the hyperinvasive clonal complexes (by definition responsible for the majority of disease) and then secondly that, if the relation were causal rather than simply clonal, there would exist within the hyperinvasive complexes an association of the element with invasive disease.

We previously identified a candidate virulence factor, the MDA prophage, by whole genome comparisons of well-characterised isolates from an epidemic situation in 1993 in the Czech Republic [10]. Here the association of the MDA phage with disease is examined in an independent data set comprising 1288 isolates from south-east England. A preliminary analysis of the results confirmed the association of the element both with disease and with certain clonal complexes, but did not permit the distinction between the possibilities that the association of the prophage with disease was due to the presence of a second virulence factor over-represented in the hyperinvasive clonal complexes, or that the prophage was one of the reasons for which the hyperinvasive clonal complexes were more likely to cause disease.

This question was addressed by evaluating the effect of the phage on the relative virulence of each clonal complex separately. The frequent horizontal genetic exchange between strains of this naturally competent species leads to genetic differences between the meningococci comprising a given clonal complex [15] and to differences in their pathogenic potential. This heterogeneity provides the opportunity to analyse the association of a candidate virulence gene with disease. In this way between 21 and 45% of the disease-causing potential of the common hyperinvasive clonal complexes could be attributed to the possession of the MDA phage, or to factors accessory to the phage (Table 2), hence supporting the hypothesis that the element acts, in concert with other virulence factors, to increase the pathogenic potential of meningococci.

As noted previously [10], the distribution of the MDA in the collection of disease isolates was not uniform by age of patient. While the prevalence of the MDA was approximately 88% in the patients aged beween 2–12 or older than 28 years, it was over-represented (93%) in the 13–28 year age band but under-represented (75%) in those patients aged less than 2 years. These findings suggest that the major part of the effect of the MDA on increased invasive potential of meningococci is manifested in young adults but not in infants. This is interesting in light of the fact that young children are relatively unprotected by circulating antibody, no longer being protected by maternally-derived antibodies and not yet having acquired natural immunity by carriage of non-virulent meningococci and other commensal *Neisseria* [16], [17]. It is possible that MDA promotes disease in immunologically mature individuals, whereas its effect is not apparent in young children who are susceptible to strains of lesser virulence lacking MDA.

The means by which the bacteriophage could increase the virulence of the host strains of meningococcus remain to be determined, in contrast to some other cases (including invasive group A streptococcal disease, cholera and haemolytic uraemic syndrome caused by *Escherichia coli*) where disease symptoms are largely determined by phage-carried toxin genes (reviewed in [18], [19]). In the case of the meningococcus, one possibility is that genomic rearrangements induced by the element might lead to changes in gene expression, hence disturbing the normal, non-pathogenic relationship between bacterium and human. This notion is in agreement with recent results from genome comparisons between pathogenic *Neisserial* strains [20] and between pathogens and carriage isolates [21].

In conclusion, comparative genomic analysis followed by verification in a large-scale epidemiolgical survey shows that the MDA phage is associated with invasiveness in meningococci in the age group of young adults. The study highlights the power of combining comparative genomics with human disease data collected in the community, particularly as an alternative to classical techniques in the study of specifically human diseases, where laboratory assays of pathogenicity correlates are not entirely representative.

## Supporting Information

Table S1 Characteristics of the meningococcal isolates used in this study
(2.33 MB DOC)

Table S2 Distribution of the MDA island in meningococci isolated from patients and carriers before (1999) and after (2001) the vaccination campaign
(0.13 MB DOC)
