# Supplementary material for: Association of a Bacteriophage with Meningococcal Disease in Young Adults
Source: PLoS One. 2008 Dec 9;3(12):e3885. doi: 10.1371/journal.pone.0003885 (PMC2587699; doi:10.1371/journal.pone.0003885)
Supplement: Table S1 — Characteristics of the meningococcal isolates used in this study (2.33 MB DOC) [file pone.0003885.s001.doc]

| **Table S1** | | |  |  |  |  |  |
| --- | --- | --- | --- | --- | --- | --- | --- |
| Characteristics of the meningococcal isolates used in this study | | | | | | | |
|  | | |  |  |  |  |  |
| Isolate number | Year of isolation | Serogroup | Sequence type | Clonal complex | Disease (1) or carrier (0) | Age of patient or carrier (years) | Presence (1) or absence (0) of MDA |
| 1 | 1999 | A | ST-7 | ST-5 cc | 1 | 2 | 0 |
| 2 | 1999 | C | ST-8 | ST-8 cc | 1 | 5 | 0 |
| 3 | 1999 | C | ST-8 | ST-8 cc | 1 | <1 | 1 |
| 4 | 1999 | C | ST-8 | ST-8 cc | 1 | 40 | 1 |
| 5 | 1999 | C | ST-8 | ST-8 cc | 1 | 20 | 1 |
| 6 | 1999 | C | ST-8 | ST-8 cc | 1 | 1 | 1 |
| 7 | 1999 | C | ST-8 | ST-8 cc | 1 | <1 | 1 |
| 8 | 1999 | C | ST-8 | ST-8 cc | 1 | 16 | 1 |
| 9 | 1999 | C | ST-8 | ST-8 cc | 1 | 12 | 1 |
| 10 | 1999 | C | ST-66 | ST-8 cc | 1 | 3 | 1 |
| 11 | 1999 | B | ST-153 | ST-8 cc | 1 | <1 | 1 |
| 12 | 1999 | C | ST-11 | ST-11 cc | 1 | 8 | 1 |
| 13 | 1999 | C | ST-11 | ST-11 cc | 1 | 6 | 1 |
| 14 | 1999 | C | ST-11 | ST-11 cc | 1 | <1 | 1 |
| 15 | 1999 | C | ST-11 | ST-11 cc | 1 | 54 | 1 |
| 16 | 1999 | C | ST-11 | ST-11 cc | 1 | 15 | 1 |
| 17 | 1999 | C | ST-11 | ST-11 cc | 1 | 25 | 1 |
| 18 | 1999 | C | ST-11 | ST-11 cc | 1 | 2 | 1 |
| 19 | 1999 | C | ST-11 | ST-11 cc | 1 | 25 | 1 |
| 20 | 1999 | C | ST-11 | ST-11 cc | 1 | 46 | 1 |
| 21 | 1999 | C | ST-11 | ST-11 cc | 1 | 47 | 1 |
| 22 | 1999 | C | ST-11 | ST-11 cc | 1 | 16 | 1 |
| 23 | 1999 | C | ST-11 | ST-11 cc | 1 | 18 | 1 |
| 24 | 1999 | C | ST-11 | ST-11 cc | 1 | 49 | 1 |
| 25 | 1999 | C | ST-11 | ST-11 cc | 1 | 39 | 1 |
| 26 | 1999 | C | ST-11 | ST-11 cc | 1 | 18 | 1 |
| 27 | 1999 | C | ST-11 | ST-11 cc | 1 | 40 | 1 |
| 28 | 1999 | C | ST-11 | ST-11 cc | 1 | 35 | 1 |
| 29 | 1999 | C | ST-11 | ST-11 cc | 1 | 2 | 1 |
| 30 | 1999 | C | ST-11 | ST-11 cc | 1 | 12 | 1 |
| 31 | 1999 | C | ST-11 | ST-11 cc | 1 | 7 | 1 |
| 32 | 1999 | C | ST-11 | ST-11 cc | 1 | 19 | 1 |
| 33 | 1999 | C | ST-11 | ST-11 cc | 1 | <1 | 1 |
| 34 | 1999 | C | ST-11 | ST-11 cc | 1 | 20 | 1 |
| 35 | 1999 | C | ST-11 | ST-11 cc | 1 | 47 | 1 |
| 36 | 1999 | C | ST-11 | ST-11 cc | 1 | 1 | 1 |
| 37 | 1999 | C | ST-11 | ST-11 cc | 1 | <1 | 1 |
| 38 | 1999 | C | ST-11 | ST-11 cc | 1 | 2 | 1 |
| 39 | 1999 | C | ST-11 | ST-11 cc | 1 | 31 | 1 |
| 40 | 1999 | C | ST-11 | ST-11 cc | 1 | 4 | 1 |
| 41 | 1999 | C | ST-11 | ST-11 cc | 1 | 1 | 1 |
| 42 | 1999 | C | ST-11 | ST-11 cc | 1 | 16 | 1 |
| 43 | 1999 | C | ST-11 | ST-11 cc | 1 | 47 | 1 |
| 44 | 1999 | C | ST-11 | ST-11 cc | 1 | 16 | 1 |
| 45 | 1999 | C | ST-11 | ST-11 cc | 1 | 17 | 1 |
| 46 | 1999 | C | ST-11 | ST-11 cc | 1 | 17 | 1 |
| 47 | 1999 | C | ST-11 | ST-11 cc | 1 | 3 | 1 |
| 48 | 1999 | C | ST-11 | ST-11 cc | 1 | 2 | 1 |
| 49 | 1999 | C | ST-11 | ST-11 cc | 1 | 51 | 1 |
| 50 | 1999 | C | ST-11 | ST-11 cc | 1 | 33 | 1 |
| 51 | 1999 | C | ST-11 | ST-11 cc | 1 | 49 | 1 |
| 52 | 1999 | C | ST-11 | ST-11 cc | 1 | 14 | 1 |
| 53 | 1999 | C | ST-11 | ST-11 cc | 1 | 41 | 1 |
| 54 | 1999 | C | ST-11 | ST-11 cc | 1 | 7 | 1 |
| 55 | 1999 | C | ST-11 | ST-11 cc | 1 | 6 | 1 |
| 56 | 1999 | C | ST-11 | ST-11 cc | 1 | 17 | 1 |
| 57 | 1999 | C | ST-11 | ST-11 cc | 1 | 6 | 1 |
| 58 | 1999 | C | ST-11 | ST-11 cc | 1 | 17 | 1 |
| 59 | 1999 | C | ST-11 | ST-11 cc | 1 | 5 | 1 |
| 60 | 1999 | C | ST-11 | ST-11 cc | 1 | 78 | 1 |
| 61 | 1999 | C | ST-11 | ST-11 cc | 1 | 29 | 1 |
| 62 | 1999 | C | ST-11 | ST-11 cc | 1 | 22 | 1 |
| 63 | 1999 | C | ST-11 | ST-11 cc | 1 | 6 | 1 |
| 64 | 1999 | C | ST-11 | ST-11 cc | 1 | <1 | 1 |
| 65 | 1999 | C | ST-11 | ST-11 cc | 1 | 20 | 1 |
| 66 | 1999 | B | ST-11 | ST-11 cc | 1 | 1 | 1 |
| 67 | 1999 | C | ST-67 | ST-11 cc | 1 | 3 | 1 |
| 68 | 1999 | C | ST-1410 | ST-11 cc | 1 | 16 | 1 |
| 69 | 1999 | C | ST-1988 | ST-11 cc | 1 | 2 | 1 |
| 70 | 1999 | C | ST-1988 | ST-11 cc | 1 | 43 | 1 |
| 71 | 1999 | C | ST-1988 | ST-11 cc | 1 | 2 | 1 |
| 72 | 1999 | C | ST-1988 | ST-11 cc | 1 | 17 | 1 |
| 73 | 1999 | C | ST-3751 | ST-11 cc | 1 | 19 | 1 |
| 74 | 1999 | C | ST-4976 | ST-11 cc | 1 | 21 | 1 |
| 75 | 1999 | C | ST-4977 | ST-11 cc | 1 | 75 | 1 |
| 76 | 1999 | B | ST-483 | ST-18 cc | 1 | 1 | 0 |
| 77 | 1999 | C | ST-4974 | ST-18 cc | 1 | 50 | 0 |
| 78 | 1999 | B | ST-18 | ST-18 cc | 1 | 19 | 0 |
| 79 | 1999 | C | ST-483 | ST-18 cc | 1 | 45 | 1 |
| 80 | 1999 | W135 | ST-184 | ST-22 cc | 1 | 2 | 0 |
| 81 | 1999 | W135 | ST-184 | ST-22 cc | 1 | 4 | 0 |
| 82 | 1999 | W135 | ST-184 | ST-22 cc | 1 | 8 | 0 |
| 83 | 1999 | W135 | ST-184 | ST-22 cc | 1 | 66 | 0 |
| 84 | 1999 | W135 | ST-184 | ST-22 cc | 1 | 75 | 0 |
| 85 | 1999 | W135 | ST-184 | ST-22 cc | 1 | <1 | 0 |
| 86 | 1999 | NG | ST-184 | ST-22 cc | 1 | 14 | 0 |
| 87 | 1999 | B | ST-184 | ST-22 cc | 1 | 2 | 0 |
| 88 | 1999 | W135 | ST-22 | ST-22 cc | 1 | 68 | 1 |
| 89 | 1999 | Y | ST-2692 | ST-23 cc | 1 | 9 | 0 |
| 90 | 1999 | Y | ST-4446 | ST-23 cc | 1 | 59 | 0 |
| 91 | 1999 | Y | ST-23 | ST-23 cc | 1 | 58 | 0 |
| 92 | 1999 | B | ST-34 | ST-32 cc | 1 | <1 | 1 |
| 93 | 1999 | B | ST-290 | ST-32 cc | 1 | 2 | 1 |
| 94 | 1999 | C | ST-32 | ST-32 cc | 1 | 52 | 1 |
| 95 | 1999 | B | ST-32 | ST-32 cc | 1 | 26 | 1 |
| 96 | 1999 | B | ST-259 | ST-32 cc | 1 | 32 | 1 |
| 97 | 1999 | B | ST-639 | ST-32 cc | 1 | 47 | 1 |
| 98 | 1999 | B | ST-1398 | ST-32 cc | 1 | <1 | 1 |
| 99 | 1999 | B | ST-4024 | ST-35 cc | 1 | <1 | 1 |
| 100 | 1999 | B | ST-41 | ST-41/44 cc | 1 | 2 | 1 |
| 101 | 1999 | B | ST-41 | ST-41/44 cc | 1 | <1 | 1 |
| 102 | 1999 | B | ST-41 | ST-41/44 cc | 1 | <1 | 0 |
| 103 | 1999 | B | ST-146 | ST-41/44 cc | 1 | 69 | 1 |
| 104 | 1999 | B | ST-340 | ST-41/44 cc | 1 | <1 | 0 |
| 105 | 1999 | B | ST-482 | ST-41/44 cc | 1 | 33 | 1 |
| 106 | 1999 | B | ST-1194 | ST-41/44 cc | 1 | 1 | 0 |
| 107 | 1999 | B | ST-1823 | ST-41/44 cc | 1 | <1 | 0 |
| 108 | 1999 | B | ST-40 | ST-41/44 cc | 1 | 16 | 1 |
| 109 | 1999 | B | ST-40 | ST-41/44 cc | 1 | 17 | 1 |
| 110 | 1999 | NG | ST-41 | ST-41/44 cc | 1 | 2 | 1 |
| 111 | 1999 | B | ST-41 | ST-41/44 cc | 1 | 1 | 1 |
| 112 | 1999 | B | ST-41 | ST-41/44 cc | 1 | 2 | 1 |
| 113 | 1999 | B | ST-41 | ST-41/44 cc | 1 | 2 | 1 |
| 114 | 1999 | B | ST-41 | ST-41/44 cc | 1 | 2 | 1 |
| 115 | 1999 | B | ST-41 | ST-41/44 cc | 1 | 4 | 0 |
| 116 | 1999 | B | ST-41 | ST-41/44 cc | 1 | 10 | 1 |
| 117 | 1999 | B | ST-41 | ST-41/44 cc | 1 | 10 | 1 |
| 118 | 1999 | B | ST-41 | ST-41/44 cc | 1 | 14 | 1 |
| 119 | 1999 | B | ST-41 | ST-41/44 cc | 1 | 17 | 1 |
| 120 | 1999 | B | ST-41 | ST-41/44 cc | 1 | 18 | 1 |
| 121 | 1999 | B | ST-41 | ST-41/44 cc | 1 | 19 | 1 |
| 122 | 1999 | B | ST-41 | ST-41/44 cc | 1 | 36 | 1 |
| 123 | 1999 | B | ST-41 | ST-41/44 cc | 1 | 39 | 1 |
| 124 | 1999 | B | ST-41 | ST-41/44 cc | 1 | 44 | 1 |
| 125 | 1999 | B | ST-41 | ST-41/44 cc | 1 | 52 | 1 |
| 126 | 1999 | B | ST-41 | ST-41/44 cc | 1 | <1 | 1 |
| 127 | 1999 | B | ST-41 | ST-41/44 cc | 1 | <1 | 1 |
| 128 | 1999 | B | ST-41 | ST-41/44 cc | 1 | <1 | 1 |
| 129 | 1999 | B | ST-136 | ST-41/44 cc | 1 | 13 | 1 |
| 130 | 1999 | B | ST-154 | ST-41/44 cc | 1 | 9 | 1 |
| 131 | 1999 | B | ST-154 | ST-41/44 cc | 1 | 12 | 1 |
| 132 | 1999 | B | ST-154 | ST-41/44 cc | 1 | 15 | 1 |
| 133 | 1999 | B | ST-154 | ST-41/44 cc | 1 | 16 | 1 |
| 134 | 1999 | B | ST-180 | ST-41/44 cc | 1 | 1 | 1 |
| 135 | 1999 | B | ST-180 | ST-41/44 cc | 1 | <1 | 1 |
| 136 | 1999 | C | ST-337 | ST-41/44 cc | 1 | 4 | 1 |
| 137 | 1999 | B | ST-340 | ST-41/44 cc | 1 | 54 | 1 |
| 138 | 1999 | B | ST-340 | ST-41/44 cc | 1 | <1 | 1 |
| 139 | 1999 | B | ST-437 | ST-41/44 cc | 1 | <1 | 1 |
| 140 | 1999 | B | ST-482 | ST-41/44 cc | 1 | <1 | 1 |
| 141 | 1999 | B | ST-571 | ST-41/44 cc | 1 | 3 | 1 |
| 142 | 1999 | B | ST-1091 | ST-41/44 cc | 1 | <1 | 1 |
| 143 | 1999 | B | ST-1097 | ST-41/44 cc | 1 | <1 | 1 |
| 144 | 1999 | NG | ST-1194 | ST-41/44 cc | 1 | 5 | 1 |
| 145 | 1999 | B | ST-1489 | ST-41/44 cc | 1 | 19 | 1 |
| 146 | 1999 | B | ST-1778 | ST-41/44 cc | 1 | <1 | 1 |
| 147 | 1999 | B | ST-2080 | ST-41/44 cc | 1 | <1 | 1 |
| 148 | 1999 | B | ST-2279 | ST-41/44 cc | 1 | <1 | 1 |
| 149 | 1999 | B | ST-2671 | ST-41/44 cc | 1 | 51 | 1 |
| 150 | 1999 | B | ST-2806 | ST-41/44 cc | 1 | 19 | 1 |
| 151 | 1999 | B | ST-2821 | ST-41/44 cc | 1 | 1 | 1 |
| 152 | 1999 | B | ST-3050 | ST-41/44 cc | 1 | 4 | 1 |
| 153 | 1999 | B | ST-4029 | ST-41/44 cc | 1 | <1 | 1 |
| 154 | 1999 | B | ST-4042 | ST-41/44 cc | 1 | 2 | 1 |
| 155 | 1999 | B | ST-4113 | ST-41/44 cc | 1 | 2 | 1 |
| 156 | 1999 | B | ST-4975 | ST-41/44 cc | 1 | 4 | 1 |
| 157 | 1999 | C | ST-60 | ST-60 cc | 1 | 22 | 0 |
| 158 | 1999 | B | ST-60 | ST-60 cc | 1 | 1 | 0 |
| 159 | 1999 | B | ST-60 | ST-60 cc | 1 | 2 | 0 |
| 160 | 1999 | B | ST-60 | ST-60 cc | 1 | <1 | 0 |
| 161 | 1999 | B | ST-60 | ST-60 cc | 1 | <1 | 0 |
| 162 | 1999 | B | ST-466 | ST-60 cc | 1 | 20 | 0 |
| 163 | 1999 | B | ST-466 | ST-60 cc | 1 | 95 | 0 |
| 164 | 1999 | B | ST-1154 | ST-60 cc | 1 | 3 | 1 |
| 165 | 1999 | B | ST-4361 | ST-162 cc | 1 | <1 | 1 |
| 166 | 1999 | Y | ST-167 | ST-167 cc | 1 | 72 | 0 |
| 167 | 1999 | B | ST-840 | ST-167 cc | 1 | 4 | 1 |
| 168 | 1999 | B | ST-213 | ST-213 cc | 1 | <1 | 0 |
| 169 | 1999 | B | ST-4360 | ST-213 cc | 1 | 19 | 1 |
| 170 | 1999 | B | ST-269 | ST-269 cc | 1 | 1 | 0 |
| 171 | 1999 | NG | ST-1195 | ST-269 cc | 1 | 18 | 1 |
| 172 | 1999 | B | ST-1195 | ST-269 cc | 1 | 9 | 0 |
| 173 | 1999 | B | ST-1195 | ST-269 cc | 1 | 43 | 1 |
| 174 | 1999 | B | ST-1214 | ST-269 cc | 1 | 4 | 1 |
| 175 | 1999 | B | ST-4023 | ST-269 cc | 1 | 58 | 1 |
| 176 | 1999 | NG | ST-4973 | ST-269 cc | 1 | 1 | 1 |
| 177 | 1999 | NG | ST-4973 | ST-269 cc | 1 | 9 | 1 |
| 178 | 1999 | B | ST-13 | ST-269 cc | 1 | 1 | 0 |
| 179 | 1999 | B | ST-269 | ST-269 cc | 1 | 4 | 1 |
| 180 | 1999 | B | ST-269 | ST-269 cc | 1 | 10 | 1 |
| 181 | 1999 | B | ST-269 | ST-269 cc | 1 | 14 | 1 |
| 182 | 1999 | B | ST-269 | ST-269 cc | 1 | 17 | 1 |
| 183 | 1999 | B | ST-269 | ST-269 cc | 1 | 19 | 1 |
| 184 | 1999 | B | ST-269 | ST-269 cc | 1 | 22 | 1 |
| 185 | 1999 | B | ST-269 | ST-269 cc | 1 | 24 | 1 |
| 186 | 1999 | B | ST-269 | ST-269 cc | 1 | 28 | 1 |
| 187 | 1999 | B | ST-269 | ST-269 cc | 1 | <1 | 1 |
| 188 | 1999 | B | ST-275 | ST-269 cc | 1 | <1 | 1 |
| 189 | 1999 | B | ST-275 | ST-269 cc | 1 | <1 | 1 |
| 190 | 1999 | C | ST-467 | ST-269 cc | 1 | 14 | 1 |
| 191 | 1999 | NG | ST-479 | ST-269 cc | 1 | 16 | 1 |
| 192 | 1999 | B | ST-1049 | ST-269 cc | 1 | 1 | 1 |
| 193 | 1999 | B | ST-1092 | ST-269 cc | 1 | 8 | 1 |
| 194 | 1999 | B | ST-1163 | ST-269 cc | 1 | 46 | 1 |
| 195 | 1999 | B | ST-1195 | ST-269 cc | 1 | 10 | 1 |
| 196 | 1999 | B | ST-1416 | ST-269 cc | 1 | 74 | 1 |
| 197 | 1999 | B | ST-4362 | ST-269 cc | 1 | 16 | 1 |
| 198 | 1999 | B | ST-2762 | ST-364 cc | 1 | 1 | 1 |
| 199 | 1999 | B | ST-461 | ST-461 cc | 1 | <1 | 0 |
| 200 | 1999 | NG | ST-1157 | ST-1157 cc | 1 | 22 | 1 |
| 201 | 1999 | B | ST-1054 | ST-nd cc | 1 | <1 | 0 |
| 202 | 1999 | C | ST-1980 | ST-nd cc | 1 | 41 | 0 |
| 203 | 1999 | B | ST-1159 | ST-nd cc | 1 | <1 | 1 |
| 204 | 1999 | NG | ST-3037 | ST-8 cc | 0 | 16 | 1 |
| 205 | 1999 | C | ST-11 | ST-11 cc | 0 | 18 | 1 |
| 206 | 1999 | C | ST-11 | ST-11 cc | 0 | 18 | 1 |
| 207 | 1999 | C | ST-11 | ST-11 cc | 0 | 18 | 1 |
| 208 | 1999 | C | ST-11 | ST-11 cc | 0 | 17 | 1 |
| 209 | 1999 | C | ST-11 | ST-11 cc | 0 | 18 | 1 |
| 210 | 1999 | C | ST-11 | ST-11 cc | 0 | 17 | 1 |
| 211 | 1999 | NG | ST-11 | ST-11 cc | 0 | 17 | 1 |
| 212 | 1999 | NG | ST-11 | ST-11 cc | 0 | 18 | 1 |
| 213 | 1999 | C | ST-1410 | ST-11 cc | 0 | 17 | 1 |
| 214 | 1999 | C | ST-1410 | ST-11 cc | 0 | 17 | 1 |
| 215 | 1999 | NG | ST-22 | ST-22 cc | 0 | 16 | 0 |
| 216 | 1999 | NG | ST-22 | ST-22 cc | 0 | 16 | 0 |
| 217 | 1999 | NG | ST-22 | ST-22 cc | 0 | 16 | 0 |
| 218 | 1999 | NG | ST-22 | ST-22 cc | 0 | 17 | 0 |
| 219 | 1999 | NG | ST-22 | ST-22 cc | 0 | 18 | 0 |
| 220 | 1999 | NG | ST-22 | ST-22 cc | 0 | 17 | 0 |
| 221 | 1999 | NG | ST-22 | ST-22 cc | 0 | 18 | 0 |
| 222 | 1999 | NG | ST-114 | ST-22 cc | 0 | 16 | 0 |
| 223 | 1999 | NG | ST-184 | ST-22 cc | 0 | 17 | 0 |
| 224 | 1999 | NG | ST-184 | ST-22 cc | 0 | 17 | 0 |
| 225 | 1999 | NG | ST-184 | ST-22 cc | 0 | 17 | 0 |
| 226 | 1999 | NG | ST-184 | ST-22 cc | 0 | 17 | 0 |
| 227 | 1999 | W135 | ST-184 | ST-22 cc | 0 | 17 | 0 |
| 228 | 1999 | W135 | ST-184 | ST-22 cc | 0 | 16 | 0 |
| 229 | 1999 | W135 | ST-184 | ST-22 cc | 0 | 17 | 0 |
| 230 | 1999 | W135 | ST-184 | ST-22 cc | 0 | 16 | 0 |
| 231 | 1999 | W135 | ST-184 | ST-22 cc | 0 | 17 | 0 |
| 232 | 1999 | W135 | ST-184 | ST-22 cc | 0 | 17 | 0 |
| 233 | 1999 | W135 | ST-184 | ST-22 cc | 0 | 18 | 0 |
| 234 | 1999 | W135 | ST-184 | ST-22 cc | 0 | 18 | 0 |
| 235 | 1999 | NG | ST-1224 | ST-22 cc | 0 | 18 | 0 |
| 236 | 1999 | NG | ST-1224 | ST-22 cc | 0 | 18 | 0 |
| 237 | 1999 | NG | ST-1426 | ST-22 cc | 0 | 16 | 0 |
| 238 | 1999 | W135 | ST-1617 | ST-22 cc | 0 | 17 | 0 |
| 239 | 1999 | W135 | ST-1617 | ST-22 cc | 0 | 17 | 0 |
| 240 | 1999 | B | ST-1641 | ST-22 cc | 0 | 17 | 0 |
| 241 | 1999 | NG | ST-1647 | ST-22 cc | 0 | 17 | 0 |
| 242 | 1999 | NG | ST-1659 | ST-22 cc | 0 | 17 | 0 |
| 243 | 1999 | W135 | ST-1659 | ST-22 cc | 0 | 17 | 0 |
| 244 | 1999 | W135 | ST-1660 | ST-22 cc | 0 | 17 | 0 |
| 245 | 1999 | W135 | ST-1661 | ST-22 cc | 0 | 19 | 0 |
| 246 | 1999 | B | ST-1667 | ST-22 cc | 0 | 16 | 0 |
| 247 | 1999 | NG | ST-1667 | ST-22 cc | 0 | 18 | 0 |
| 248 | 1999 | W135 | ST-1667 | ST-22 cc | 0 | 17 | 0 |
| 249 | 1999 | W135 | ST-1667 | ST-22 cc | 0 | 17 | 0 |
| 250 | 1999 | W135 | ST-1667 | ST-22 cc | 0 | 17 | 0 |
| 251 | 1999 | W135 | ST-1667 | ST-22 cc | 0 | 18 | 0 |
| 252 | 1999 | W135 | ST-1673 | ST-22 cc | 0 | 17 | 0 |
| 253 | 1999 | W135 | ST-1673 | ST-22 cc | 0 | 19 | 0 |
| 254 | 1999 | NG | ST-1674 | ST-22 cc | 0 | 16 | 0 |
| 255 | 1999 | Y | ST-2180 | ST-22 cc | 0 | 17 | 0 |
| 256 | 1999 | Y | ST-2180 | ST-22 cc | 0 | 17 | 0 |
| 257 | 1999 | W135 | ST-2451 | ST-22 cc | 0 | 16 | 0 |
| 258 | 1999 | NG | ST-3059 | ST-22 cc | 0 | 17 | 0 |
| 259 | 1999 | W135 | ST-3137 | ST-22 cc | 0 | 17 | 0 |
| 260 | 1999 | W135 | ST-3159 | ST-22 cc | 0 | 16 | 0 |
| 261 | 1999 | NG | ST-3182 | ST-22 cc | 0 | 17 | 0 |
| 262 | 1999 | NG | ST-3227 | ST-22 cc | 0 | 16 | 0 |
| 263 | 1999 | Y | ST-114 | ST-22 cc | 0 | 16 | 1 |
| 264 | 1999 | NG | ST-184 | ST-22 cc | 0 | 16 | 0 |
| 265 | 1999 | W135 | ST-184 | ST-22 cc | 0 | 17 | 0 |
| 266 | 1999 | NG | ST-1224 | ST-22 cc | 0 | 18 | 1 |
| 267 | 1999 | NG | ST-1440 | ST-22 cc | 0 | 16 | 1 |
| 268 | 1999 | NG | ST-1449 | ST-22 cc | 0 | 16 | 1 |
| 269 | 1999 | Y | ST-2420 | ST-22 cc | 0 | 17 | 1 |
| 270 | 1999 | NG | ST-23 | ST-23 cc | 0 | 17 | 0 |
| 271 | 1999 | NG | ST-23 | ST-23 cc | 0 | 16 | 0 |
| 272 | 1999 | Y | ST-23 | ST-23 cc | 0 | 16 | 0 |
| 273 | 1999 | Y | ST-23 | ST-23 cc | 0 | 17 | 0 |
| 274 | 1999 | Y | ST-23 | ST-23 cc | 0 | 18 | 0 |
| 275 | 1999 | NG | ST-1655 | ST-23 cc | 0 | 17 | 0 |
| 276 | 1999 | Y | ST-1655 | ST-23 cc | 0 | 17 | 0 |
| 277 | 1999 | Y | ST-1655 | ST-23 cc | 0 | 19 | 0 |
| 278 | 1999 | Y | ST-1664 | ST-23 cc | 0 | 16 | 0 |
| 279 | 1999 | Y | ST-3228 | ST-23 cc | 0 | 17 | 0 |
| 280 | 1999 | NG | ST-23 | ST-23 cc | 0 | 17 | 1 |
| 281 | 1999 | NG | ST-23 | ST-23 cc | 0 | 17 | 1 |
| 282 | 1999 | NG | ST-23 | ST-23 cc | 0 | 16 | 1 |
| 283 | 1999 | NG | ST-23 | ST-23 cc | 0 | 17 | 1 |
| 284 | 1999 | Y | ST-23 | ST-23 cc | 0 | 16 | 1 |
| 285 | 1999 | Y | ST-23 | ST-23 cc | 0 | 17 | 1 |
| 286 | 1999 | Y | ST-23 | ST-23 cc | 0 | 17 | 1 |
| 287 | 1999 | Y | ST-23 | ST-23 cc | 0 | 17 | 1 |
| 288 | 1999 | Y | ST-23 | ST-23 cc | 0 | 16 | 1 |
| 289 | 1999 | Y | ST-23 | ST-23 cc | 0 | 18 | 1 |
| 290 | 1999 | Y | ST-23 | ST-23 cc | 0 | 18 | 1 |
| 291 | 1999 | Y | ST-23 | ST-23 cc | 0 | 16 | 1 |
| 292 | 1999 | Y | ST-23 | ST-23 cc | 0 | 17 | 1 |
| 293 | 1999 | Y | ST-23 | ST-23 cc | 0 | 16 | 1 |
| 294 | 1999 | Y | ST-23 | ST-23 cc | 0 | 17 | 1 |
| 295 | 1999 | Y | ST-1448 | ST-23 cc | 0 | 17 | 1 |
| 296 | 1999 | Y | ST-1625 | ST-23 cc | 0 | 17 | 1 |
| 297 | 1999 | Y | ST-1652 | ST-23 cc | 0 | 18 | 1 |
| 298 | 1999 | Y | ST-1657 | ST-23 cc | 0 | 18 | 1 |
| 299 | 1999 | Y | ST-3158 | ST-23 cc | 0 | 17 | 1 |
| 300 | 1999 | Y | ST-3171 | ST-23 cc | 0 | 16 | 1 |
| 301 | 1999 | Y | ST-3184 | ST-23 cc | 0 | 17 | 1 |
| 302 | 1999 | NG | ST-3187 | ST-23 cc | 0 | 17 | 1 |
| 303 | 1999 | Y | ST-4387 | ST-23 cc | 0 | 16 | 1 |
| 304 | 1999 | B | ST-32 | ST-32 cc | 0 | 16 | 1 |
| 305 | 1999 | NG | ST-33 | ST-32 cc | 0 | 18 | 1 |
| 306 | 1999 | NG | ST-34 | ST-32 cc | 0 | 17 | 1 |
| 307 | 1999 | B | ST-1680 | ST-32 cc | 0 | 18 | 1 |
| 308 | 1999 | NG | ST-2400 | ST-32 cc | 0 | 16 | 1 |
| 309 | 1999 | 29E | ST-2472 | ST-32 cc | 0 | 17 | 1 |
| 310 | 1999 | B | ST-1441 | ST-35 cc | 0 | 17 | 0 |
| 311 | 1999 | B | ST-3177 | ST-35 cc | 0 | 16 | 0 |
| 312 | 1999 | B | ST-35 | ST-35 cc | 0 | 17 | 1 |
| 313 | 1999 | NG | ST-278 | ST-35 cc | 0 | 16 | 1 |
| 314 | 1999 | B | ST-457 | ST-35 cc | 0 | 16 | 1 |
| 315 | 1999 | NG | ST-457 | ST-35 cc | 0 | 16 | 1 |
| 316 | 1999 | 29E | ST-1417 | ST-35 cc | 0 | 16 | 1 |
| 317 | 1999 | C | ST-1679 | ST-35 cc | 0 | 17 | 1 |
| 318 | 1999 | NG | ST-2370 | ST-35 cc | 0 | 16 | 1 |
| 319 | 1999 | NG | ST-2381 | ST-35 cc | 0 | 17 | 1 |
| 320 | 1999 | NG | ST-2422 | ST-35 cc | 0 | 16 | 1 |
| 321 | 1999 | B | ST-2463 | ST-35 cc | 0 | 18 | 1 |
| 322 | 1999 | B | ST-3073 | ST-35 cc | 0 | 16 | 1 |
| 323 | 1999 | B | ST-3074 | ST-35 cc | 0 | 16 | 1 |
| 324 | 1999 | Y | ST-3075 | ST-35 cc | 0 | 16 | 1 |
| 325 | 1999 | B | ST-3078 | ST-35 cc | 0 | 17 | 1 |
| 326 | 1999 | B | ST-43 | ST-41/44 cc | 0 | 16 | 0 |
| 327 | 1999 | B | ST-43 | ST-41/44 cc | 0 | 16 | 0 |
| 328 | 1999 | B | ST-170 | ST-41/44 cc | 0 | 17 | 0 |
| 329 | 1999 | 29E | ST-41 | ST-41/44 cc | 0 | 17 | 1 |
| 330 | 1999 | B | ST-41 | ST-41/44 cc | 0 | 17 | 1 |
| 331 | 1999 | B | ST-41 | ST-41/44 cc | 0 | 16 | 1 |
| 332 | 1999 | B | ST-41 | ST-41/44 cc | 0 | 17 | 1 |
| 333 | 1999 | NG | ST-41 | ST-41/44 cc | 0 | 17 | 1 |
| 334 | 1999 | NG | ST-41 | ST-41/44 cc | 0 | 17 | 1 |
| 335 | 1999 | NG | ST-41 | ST-41/44 cc | 0 | 17 | 1 |
| 336 | 1999 | NG | ST-41 | ST-41/44 cc | 0 | 18 | 1 |
| 337 | 1999 | B | ST-43 | ST-41/44 cc | 0 | 17 | 1 |
| 338 | 1999 | B | ST-43 | ST-41/44 cc | 0 | 17 | 1 |
| 339 | 1999 | B | ST-43 | ST-41/44 cc | 0 | 17 | 1 |
| 340 | 1999 | B | ST-43 | ST-41/44 cc | 0 | 16 | 1 |
| 341 | 1999 | B | ST-43 | ST-41/44 cc | 0 | 17 | 1 |
| 342 | 1999 | 29E | ST-44 | ST-41/44 cc | 0 | 17 | 1 |
| 343 | 1999 | NG | ST-44 | ST-41/44 cc | 0 | 17 | 1 |
| 344 | 1999 | B | ST-136 | ST-41/44 cc | 0 | 17 | 1 |
| 345 | 1999 | B | ST-136 | ST-41/44 cc | 0 | 17 | 1 |
| 346 | 1999 | B | ST-136 | ST-41/44 cc | 0 | 17 | 1 |
| 347 | 1999 | B | ST-180 | ST-41/44 cc | 0 | 17 | 1 |
| 348 | 1999 | B | ST-180 | ST-41/44 cc | 0 | 18 | 1 |
| 349 | 1999 | B | ST-180 | ST-41/44 cc | 0 | 17 | 1 |
| 350 | 1999 | B | ST-180 | ST-41/44 cc | 0 | 16 | 1 |
| 351 | 1999 | B | ST-180 | ST-41/44 cc | 0 | 16 | 1 |
| 352 | 1999 | B | ST-180 | ST-41/44 cc | 0 | 16 | 1 |
| 353 | 1999 | NG | ST-409 | ST-41/44 cc | 0 | 17 | 1 |
| 354 | 1999 | NG | ST-577 | ST-41/44 cc | 0 | 17 | 1 |
| 355 | 1999 | NG | ST-833 | ST-41/44 cc | 0 | 17 | 1 |
| 356 | 1999 | B | ST-1097 | ST-41/44 cc | 0 | 16 | 1 |
| 357 | 1999 | B | ST-1097 | ST-41/44 cc | 0 | 18 | 1 |
| 358 | 1999 | B | ST-1097 | ST-41/44 cc | 0 | 17 | 1 |
| 359 | 1999 | B | ST-1097 | ST-41/44 cc | 0 | 18 | 1 |
| 360 | 1999 | B | ST-1097 | ST-41/44 cc | 0 | 18 | 1 |
| 361 | 1999 | B | ST-1097 | ST-41/44 cc | 0 | 17 | 1 |
| 362 | 1999 | B | ST-1097 | ST-41/44 cc | 0 | 16 | 1 |
| 363 | 1999 | NG | ST-1228 | ST-41/44 cc | 0 | 17 | 1 |
| 364 | 1999 | B | ST-1413 | ST-41/44 cc | 0 | 16 | 1 |
| 365 | 1999 | B | ST-1414 | ST-41/44 cc | 0 | 16 | 1 |
| 366 | 1999 | B | ST-1414 | ST-41/44 cc | 0 | 18 | 1 |
| 367 | 1999 | B | ST-1415 | ST-41/44 cc | 0 | 16 | 1 |
| 368 | 1999 | B | ST-1415 | ST-41/44 cc | 0 | 16 | 1 |
| 369 | 1999 | B | ST-1415 | ST-41/44 cc | 0 | 16 | 1 |
| 370 | 1999 | B | ST-1423 | ST-41/44 cc | 0 | 16 | 1 |
| 371 | 1999 | B | ST-1423 | ST-41/44 cc | 0 | 18 | 1 |
| 372 | 1999 | NG | ST-1425 | ST-41/44 cc | 0 | 17 | 1 |
| 373 | 1999 | B | ST-1432 | ST-41/44 cc | 0 | 16 | 1 |
| 374 | 1999 | 29E | ST-1437 | ST-41/44 cc | 0 | 18 | 1 |
| 375 | 1999 | B | ST-1439 | ST-41/44 cc | 0 | 16 | 0 |
| 376 | 1999 | B | ST-1443 | ST-41/44 cc | 0 | 16 | 1 |
| 377 | 1999 | NG | ST-1645 | ST-41/44 cc | 0 | 16 | 1 |
| 378 | 1999 | NG | ST-1656 | ST-41/44 cc | 0 | 16 | 1 |
| 379 | 1999 | B | ST-1676 | ST-41/44 cc | 0 | 17 | 1 |
| 380 | 1999 | B | ST-2364 | ST-41/44 cc | 0 | 18 | 1 |
| 381 | 1999 | NG | ST-2406 | ST-41/44 cc | 0 | 17 | 1 |
| 382 | 1999 | NG | ST-2416 | ST-41/44 cc | 0 | 18 | 1 |
| 383 | 1999 | C | ST-3038 | ST-41/44 cc | 0 | 18 | 1 |
| 384 | 1999 | B | ST-3046 | ST-41/44 cc | 0 | 17 | 1 |
| 385 | 1999 | B | ST-3160 | ST-41/44 cc | 0 | 17 | 1 |
| 386 | 1999 | B | ST-3161 | ST-41/44 cc | 0 | 17 | 1 |
| 387 | 1999 | 29E | ST-53 | ST-53 cc | 0 | 17 | 0 |
| 388 | 1999 | 29E | ST-53 | ST-53 cc | 0 | 17 | 0 |
| 389 | 1999 | 29E | ST-53 | ST-53 cc | 0 | 17 | 0 |
| 390 | 1999 | 29E | ST-53 | ST-53 cc | 0 | 17 | 0 |
| 391 | 1999 | 29E | ST-53 | ST-53 cc | 0 | 18 | 0 |
| 392 | 1999 | NG | ST-53 | ST-53 cc | 0 | 17 | 0 |
| 393 | 1999 | NG | ST-53 | ST-53 cc | 0 | 17 | 0 |
| 394 | 1999 | NG | ST-53 | ST-53 cc | 0 | 16 | 0 |
| 395 | 1999 | NG | ST-53 | ST-53 cc | 0 | 17 | 0 |
| 396 | 1999 | NG | ST-53 | ST-53 cc | 0 | 16 | 0 |
| 397 | 1999 | NG | ST-53 | ST-53 cc | 0 | 16 | 0 |
| 398 | 1999 | NG | ST-53 | ST-53 cc | 0 | 17 | 0 |
| 399 | 1999 | NG | ST-53 | ST-53 cc | 0 | 16 | 0 |
| 400 | 1999 | NG | ST-53 | ST-53 cc | 0 | 17 | 0 |
| 401 | 1999 | NG | ST-53 | ST-53 cc | 0 | 17 | 0 |
| 402 | 1999 | NG | ST-53 | ST-53 cc | 0 | 16 | 0 |
| 403 | 1999 | NG | ST-53 | ST-53 cc | 0 | 17 | 0 |
| 404 | 1999 | NG | ST-53 | ST-53 cc | 0 | 17 | 0 |
| 405 | 1999 | NG | ST-53 | ST-53 cc | 0 | 16 | 0 |
| 406 | 1999 | NG | ST-53 | ST-53 cc | 0 | 17 | 0 |
| 407 | 1999 | NG | ST-53 | ST-53 cc | 0 | 17 | 0 |
| 408 | 1999 | NG | ST-53 | ST-53 cc | 0 | 17 | 0 |
| 409 | 1999 | NG | ST-53 | ST-53 cc | 0 | 17 | 0 |
| 410 | 1999 | W135 | ST-53 | ST-53 cc | 0 | 16 | 0 |
| 411 | 1999 | NG | ST-1266 | ST-53 cc | 0 | 16 | 0 |
| 412 | 1999 | NG | ST-1452 | ST-53 cc | 0 | 17 | 0 |
| 413 | 1999 | NG | ST-1654 | ST-53 cc | 0 | 16 | 0 |
| 414 | 1999 | NG | ST-1663 | ST-53 cc | 0 | 16 | 0 |
| 415 | 1999 | NG | ST-2124 | ST-53 cc | 0 | 16 | 0 |
| 416 | 1999 | NG | ST-2312 | ST-53 cc | 0 | 17 | 0 |
| 417 | 1999 | 29E | ST-2361 | ST-53 cc | 0 | 17 | 0 |
| 418 | 1999 | NG | ST-2423 | ST-53 cc | 0 | 16 | 0 |
| 419 | 1999 | 29E | ST-2438 | ST-53 cc | 0 | 16 | 0 |
| 420 | 1999 | NG | ST-2464 | ST-53 cc | 0 | 17 | 0 |
| 421 | 1999 | NG | ST-3178 | ST-53 cc | 0 | 17 | 0 |
| 422 | 1999 | NG | ST-3178 | ST-53 cc | 0 | 17 | 0 |
| 423 | 1999 | NG | ST-3219 | ST-53 cc | 0 | 16 | 0 |
| 424 | 1999 | NG | ST-3229 | ST-53 cc | 0 | 18 | 0 |
| 425 | 1999 | 29E | ST-60 | ST-60 cc | 0 | 16 | 0 |
| 426 | 1999 | 29E | ST-60 | ST-60 cc | 0 | 16 | 0 |
| 427 | 1999 | 29E | ST-60 | ST-60 cc | 0 | 18 | 0 |
| 428 | 1999 | 29E | ST-60 | ST-60 cc | 0 | 17 | 0 |
| 429 | 1999 | 29E | ST-60 | ST-60 cc | 0 | 17 | 0 |
| 430 | 1999 | 29E | ST-60 | ST-60 cc | 0 | 16 | 0 |
| 431 | 1999 | 29E | ST-60 | ST-60 cc | 0 | 16 | 0 |
| 432 | 1999 | 29E | ST-60 | ST-60 cc | 0 | 18 | 0 |
| 433 | 1999 | 29E | ST-60 | ST-60 cc | 0 | 17 | 0 |
| 434 | 1999 | 29E | ST-60 | ST-60 cc | 0 | 17 | 0 |
| 435 | 1999 | 29E | ST-60 | ST-60 cc | 0 | 16 | 0 |
| 436 | 1999 | 29E | ST-60 | ST-60 cc | 0 | 16 | 0 |
| 437 | 1999 | NG | ST-60 | ST-60 cc | 0 | 18 | 0 |
| 438 | 1999 | NG | ST-60 | ST-60 cc | 0 | 16 | 0 |
| 439 | 1999 | NG | ST-60 | ST-60 cc | 0 | 17 | 0 |
| 440 | 1999 | NG | ST-60 | ST-60 cc | 0 | 16 | 0 |
| 441 | 1999 | NG | ST-60 | ST-60 cc | 0 | 16 | 0 |
| 442 | 1999 | 29E | ST-466 | ST-60 cc | 0 | 18 | 0 |
| 443 | 1999 | 29E | ST-913 | ST-60 cc | 0 | 17 | 0 |
| 444 | 1999 | 29E | ST-1383 | ST-60 cc | 0 | 17 | 0 |
| 445 | 1999 | 29E | ST-1411 | ST-60 cc | 0 | 16 | 0 |
| 446 | 1999 | NG | ST-1429 | ST-60 cc | 0 | 16 | 0 |
| 447 | 1999 | 29E | ST-1430 | ST-60 cc | 0 | 17 | 0 |
| 448 | 1999 | 29E | ST-1453 | ST-60 cc | 0 | 16 | 0 |
| 449 | 1999 | Y | ST-1637 | ST-60 cc | 0 | 18 | 0 |
| 450 | 1999 | 29E | ST-1650 | ST-60 cc | 0 | 16 | 0 |
| 451 | 1999 | 29E | ST-1658 | ST-60 cc | 0 | 18 | 0 |
| 452 | 1999 | B | ST-1670 | ST-60 cc | 0 | 19 | 0 |
| 453 | 1999 | NG | ST-1677 | ST-60 cc | 0 | 16 | 0 |
| 454 | 1999 | 29E | ST-2452 | ST-60 cc | 0 | 16 | 0 |
| 455 | 1999 | 29E | ST-2453 | ST-60 cc | 0 | 18 | 0 |
| 456 | 1999 | 29E | ST-2454 | ST-60 cc | 0 | 20 | 0 |
| 457 | 1999 | NG | ST-2456 | ST-60 cc | 0 | 19 | 0 |
| 458 | 1999 | NG | ST-3807 | ST-60 cc | 0 | 16 | 0 |
| 459 | 1999 | 29E | ST-60 | ST-60 cc | 0 | 16 | 1 |
| 460 | 1999 | 29E | ST-60 | ST-60 cc | 0 | 17 | 1 |
| 461 | 1999 | 29E | ST-60 | ST-60 cc | 0 | 16 | 1 |
| 462 | 1999 | 29E | ST-1430 | ST-60 cc | 0 | 17 | 1 |
| 463 | 1999 | B | ST-1640 | ST-60 cc | 0 | 18 | 1 |
| 464 | 1999 | NG | ST-3231 | ST-60 cc | 0 | 17 | 1 |
| 465 | 1999 | B | ST-103 | ST-103 cc | 0 | 18 | 0 |
| 466 | 1999 | NG | ST-103 | ST-103 cc | 0 | 17 | 0 |
| 467 | 1999 | NG | ST-103 | ST-103 cc | 0 | 16 | 0 |
| 468 | 1999 | Z | ST-103 | ST-103 cc | 0 | 18 | 0 |
| 469 | 1999 | Z | ST-103 | ST-103 cc | 0 | 17 | 0 |
| 470 | 1999 | NG | ST-1418 | ST-103 cc | 0 | 17 | 0 |
| 471 | 1999 | NG | ST-1418 | ST-103 cc | 0 | 17 | 0 |
| 472 | 1999 | 29E | ST-103 | ST-103 cc | 0 | 16 | 1 |
| 473 | 1999 | 29E | ST-103 | ST-103 cc | 0 | 18 | 1 |
| 474 | 1999 | B | ST-1418 | ST-103 cc | 0 | 17 | 1 |
| 475 | 1999 | B | ST-162 | ST-162 cc | 0 | 16 | 1 |
| 476 | 1999 | B | ST-162 | ST-162 cc | 0 | 16 | 1 |
| 477 | 1999 | B | ST-162 | ST-162 cc | 0 | 16 | 1 |
| 478 | 1999 | B | ST-162 | ST-162 cc | 0 | 18 | 1 |
| 479 | 1999 | B | ST-2353 | ST-162 cc | 0 | 16 | 1 |
| 480 | 1999 | B | ST-3027 | ST-162 cc | 0 | 17 | 1 |
| 481 | 1999 | B | ST-3029 | ST-162 cc | 0 | 16 | 1 |
| 482 | 1999 | B | ST-3032 | ST-162 cc | 0 | 17 | 1 |
| 483 | 1999 | NG | ST-167 | ST-167 cc | 0 | 18 | 0 |
| 484 | 1999 | NG | ST-167 | ST-167 cc | 0 | 17 | 0 |
| 485 | 1999 | NG | ST-168 | ST-167 cc | 0 | 18 | 0 |
| 486 | 1999 | NG | ST-168 | ST-167 cc | 0 | 16 | 0 |
| 487 | 1999 | NG | ST-168 | ST-167 cc | 0 | 16 | 0 |
| 488 | 1999 | NG | ST-168 | ST-167 cc | 0 | 18 | 0 |
| 489 | 1999 | NG | ST-168 | ST-167 cc | 0 | 16 | 0 |
| 490 | 1999 | Y | ST-279 | ST-167 cc | 0 | 17 | 0 |
| 491 | 1999 | Y | ST-279 | ST-167 cc | 0 | 16 | 0 |
| 492 | 1999 | NG | ST-766 | ST-167 cc | 0 | 16 | 0 |
| 493 | 1999 | NG | ST-1412 | ST-167 cc | 0 | 17 | 0 |
| 494 | 1999 | NG | ST-1412 | ST-167 cc | 0 | 17 | 0 |
| 495 | 1999 | B | ST-1436 | ST-167 cc | 0 | 18 | 0 |
| 496 | 1999 | NG | ST-1624 | ST-167 cc | 0 | 16 | 0 |
| 497 | 1999 | NG | ST-1627 | ST-167 cc | 0 | 16 | 0 |
| 498 | 1999 | NG | ST-1636 | ST-167 cc | 0 | 17 | 0 |
| 499 | 1999 | NG | ST-1636 | ST-167 cc | 0 | 17 | 0 |
| 500 | 1999 | Y | ST-1636 | ST-167 cc | 0 | 18 | 0 |
| 501 | 1999 | Y | ST-1636 | ST-167 cc | 0 | 17 | 0 |
| 502 | 1999 | NG | ST-168 | ST-167 cc | 0 | 16 | 1 |
| 503 | 1999 | Y | ST-168 | ST-167 cc | 0 | 17 | 1 |
| 504 | 1999 | Y | ST-279 | ST-167 cc | 0 | 16 | 0 |
| 505 | 1999 | Y | ST-279 | ST-167 cc | 0 | 18 | 0 |
| 506 | 1999 | B | ST-1643 | ST-167 cc | 0 | 17 | 1 |
| 507 | 1999 | NG | ST-39 | ST-198 cc | 0 | 17 | 0 |
| 508 | 1999 | 29E | ST-198 | ST-198 cc | 0 | 18 | 0 |
| 509 | 1999 | NG | ST-198 | ST-198 cc | 0 | 17 | 0 |
| 510 | 1999 | NG | ST-198 | ST-198 cc | 0 | 16 | 0 |
| 511 | 1999 | NG | ST-198 | ST-198 cc | 0 | 17 | 0 |
| 512 | 1999 | NG | ST-198 | ST-198 cc | 0 | 17 | 0 |
| 513 | 1999 | NG | ST-198 | ST-198 cc | 0 | 16 | 0 |
| 514 | 1999 | NG | ST-198 | ST-198 cc | 0 | 16 | 0 |
| 515 | 1999 | NG | ST-198 | ST-198 cc | 0 | 17 | 0 |
| 516 | 1999 | NG | ST-198 | ST-198 cc | 0 | 17 | 0 |
| 517 | 1999 | NG | ST-198 | ST-198 cc | 0 | 16 | 0 |
| 518 | 1999 | NG | ST-198 | ST-198 cc | 0 | 16 | 0 |
| 519 | 1999 | NG | ST-823 | ST-198 cc | 0 | 17 | 0 |
| 520 | 1999 | NG | ST-823 | ST-198 cc | 0 | 16 | 0 |
| 521 | 1999 | NG | ST-823 | ST-198 cc | 0 | 17 | 0 |
| 522 | 1999 | NG | ST-1956 | ST-198 cc | 0 | 17 | 0 |
| 523 | 1999 | NG | ST-2382 | ST-198 cc | 0 | 17 | 0 |
| 524 | 1999 | NG | ST-1681 | ST-198 cc | 0 | 17 | 1 |
| 525 | 1999 | B | ST-213 | ST-213 cc | 0 | 16 | 0 |
| 526 | 1999 | B | ST-213 | ST-213 cc | 0 | 16 | 0 |
| 527 | 1999 | B | ST-213 | ST-213 cc | 0 | 17 | 0 |
| 528 | 1999 | B | ST-213 | ST-213 cc | 0 | 16 | 0 |
| 529 | 1999 | B | ST-213 | ST-213 cc | 0 | 19 | 0 |
| 530 | 1999 | B | ST-213 | ST-213 cc | 0 | 17 | 0 |
| 531 | 1999 | B | ST-213 | ST-213 cc | 0 | 17 | 0 |
| 532 | 1999 | B | ST-213 | ST-213 cc | 0 | 16 | 0 |
| 533 | 1999 | B | ST-213 | ST-213 cc | 0 | 16 | 0 |
| 534 | 1999 | B | ST-213 | ST-213 cc | 0 | 16 | 0 |
| 535 | 1999 | B | ST-213 | ST-213 cc | 0 | 17 | 0 |
| 536 | 1999 | B | ST-213 | ST-213 cc | 0 | 18 | 0 |
| 537 | 1999 | B | ST-213 | ST-213 cc | 0 | 19 | 0 |
| 538 | 1999 | NG | ST-213 | ST-213 cc | 0 | 16 | 0 |
| 539 | 1999 | NG | ST-213 | ST-213 cc | 0 | 16 | 0 |
| 540 | 1999 | NG | ST-213 | ST-213 cc | 0 | 16 | 0 |
| 541 | 1999 | B | ST-1447 | ST-213 cc | 0 | 17 | 0 |
| 542 | 1999 | B | ST-1631 | ST-213 cc | 0 | 17 | 0 |
| 543 | 1999 | B | ST-1638 | ST-213 cc | 0 | 17 | 0 |
| 544 | 1999 | B | ST-1644 | ST-213 cc | 0 | 18 | 0 |
| 545 | 1999 | B | ST-3113 | ST-213 cc | 0 | 17 | 0 |
| 546 | 1999 | B | ST-3273 | ST-213 cc | 0 | 17 | 0 |
| 547 | 1999 | NG | ST-1458 | ST-213 cc | 0 | 17 | 1 |
| 548 | 1999 | NG | ST-254 | ST-254 cc | 0 | 17 | 0 |
| 549 | 1999 | NG | ST-254 | ST-254 cc | 0 | 17 | 0 |
| 550 | 1999 | NG | ST-1827 | ST-254 cc | 0 | 16 | 0 |
| 551 | 1999 | 29E | ST-2360 | ST-254 cc | 0 | 16 | 0 |
| 552 | 1999 | NG | ST-3147 | ST-254 cc | 0 | 16 | 0 |
| 553 | 1999 | 29E | ST-4134 | ST-254 cc | 0 | 18 | 0 |
| 554 | 1999 | NG | ST-254 | ST-254 cc | 0 | 17 | 1 |
| 555 | 1999 | B | ST-269 | ST-269 cc | 0 | 16 | 1 |
| 556 | 1999 | NG | ST-1438 | ST-269 cc | 0 | 16 | 0 |
| 557 | 1999 | B | ST-269 | ST-269 cc | 0 | 16 | 1 |
| 558 | 1999 | B | ST-269 | ST-269 cc | 0 | 16 | 1 |
| 559 | 1999 | B | ST-269 | ST-269 cc | 0 | 18 | 1 |
| 560 | 1999 | B | ST-269 | ST-269 cc | 0 | 16 | 1 |
| 561 | 1999 | NG | ST-269 | ST-269 cc | 0 | 16 | 1 |
| 562 | 1999 | NG | ST-269 | ST-269 cc | 0 | 16 | 1 |
| 563 | 1999 | NG | ST-269 | ST-269 cc | 0 | 17 | 1 |
| 564 | 1999 | NG | ST-269 | ST-269 cc | 0 | 17 | 1 |
| 565 | 1999 | NG | ST-269 | ST-269 cc | 0 | 16 | 1 |
| 566 | 1999 | NG | ST-269 | ST-269 cc | 0 | 16 | 1 |
| 567 | 1999 | NG | ST-269 | ST-269 cc | 0 | 16 | 1 |
| 568 | 1999 | NG | ST-269 | ST-269 cc | 0 | 17 | 1 |
| 569 | 1999 | B | ST-275 | ST-269 cc | 0 | 17 | 1 |
| 570 | 1999 | B | ST-275 | ST-269 cc | 0 | 17 | 1 |
| 571 | 1999 | B | ST-275 | ST-269 cc | 0 | 17 | 1 |
| 572 | 1999 | NG | ST-275 | ST-269 cc | 0 | 17 | 1 |
| 573 | 1999 | NG | ST-275 | ST-269 cc | 0 | 17 | 1 |
| 574 | 1999 | NG | ST-275 | ST-269 cc | 0 | 16 | 1 |
| 575 | 1999 | NG | ST-275 | ST-269 cc | 0 | 16 | 1 |
| 576 | 1999 | NG | ST-275 | ST-269 cc | 0 | 16 | 1 |
| 577 | 1999 | B | ST-283 | ST-269 cc | 0 | 17 | 1 |
| 578 | 1999 | C | ST-467 | ST-269 cc | 0 | 16 | 1 |
| 579 | 1999 | NG | ST-467 | ST-269 cc | 0 | 16 | 1 |
| 580 | 1999 | B | ST-1163 | ST-269 cc | 0 | 17 | 1 |
| 581 | 1999 | B | ST-1416 | ST-269 cc | 0 | 18 | 1 |
| 582 | 1999 | NG | ST-1682 | ST-269 cc | 0 | 18 | 1 |
| 583 | 1999 | NG | ST-2369 | ST-269 cc | 0 | 17 | 1 |
| 584 | 1999 | B | ST-3064 | ST-269 cc | 0 | 17 | 1 |
| 585 | 1999 | NG | ST-3069 | ST-269 cc | 0 | 17 | 1 |
| 586 | 1999 | B | ST-1454 | ST-334 cc | 0 | 16 | 0 |
| 587 | 1999 | B | ST-1666 | ST-364 cc | 0 | 17 | 0 |
| 588 | 1999 | X | ST-750 | ST-750 cc | 0 | 18 | 1 |
| 589 | 1999 | X | ST-750 | ST-750 cc | 0 | 17 | 1 |
| 590 | 1999 | X | ST-750 | ST-750 cc | 0 | 16 | 1 |
| 591 | 1999 | X | ST-750 | ST-750 cc | 0 | 17 | 1 |
| 592 | 1999 | X | ST-2355 | ST-750 cc | 0 | 16 | 1 |
| 593 | 1999 | NG | ST-1445 | ST-865 cc | 0 | 18 | 0 |
| 594 | 1999 | NG | ST-1675 | ST-865 cc | 0 | 17 | 0 |
| 595 | 1999 | NG | ST-3240 | ST-865 cc | 0 | 17 | 0 |
| 596 | 1999 | NG | ST-1157 | ST-1157 cc | 0 | 17 | 0 |
| 597 | 1999 | NG | ST-1157 | ST-1157 cc | 0 | 16 | 0 |
| 598 | 1999 | NG | ST-1157 | ST-1157 cc | 0 | 18 | 0 |
| 599 | 1999 | 29E | ST-1157 | ST-1157 cc | 0 | 16 | 1 |
| 600 | 1999 | 29E | ST-1157 | ST-1157 cc | 0 | 17 | 1 |
| 601 | 1999 | 29E | ST-1157 | ST-1157 cc | 0 | 17 | 1 |
| 602 | 1999 | NG | ST-1157 | ST-1157 cc | 0 | 18 | 1 |
| 603 | 1999 | NG | ST-1157 | ST-1157 cc | 0 | 17 | 1 |
| 604 | 1999 | NG | ST-1157 | ST-1157 cc | 0 | 16 | 1 |
| 605 | 1999 | NG | ST-1157 | ST-1157 cc | 0 | 17 | 1 |
| 606 | 1999 | NG | ST-1157 | ST-1157 cc | 0 | 16 | 1 |
| 607 | 1999 | NG | ST-1157 | ST-1157 cc | 0 | 17 | 1 |
| 608 | 1999 | NG | ST-1157 | ST-1157 cc | 0 | 18 | 1 |
| 609 | 1999 | NG | ST-1157 | ST-1157 cc | 0 | 17 | 1 |
| 610 | 1999 | NG | ST-1157 | ST-1157 cc | 0 | 17 | 1 |
| 611 | 1999 | NG | ST-1157 | ST-1157 cc | 0 | 17 | 1 |
| 612 | 1999 | NG | ST-1157 | ST-1157 cc | 0 | 17 | 1 |
| 613 | 1999 | NG | ST-1157 | ST-1157 cc | 0 | 16 | 1 |
| 614 | 1999 | NG | ST-1157 | ST-1157 cc | 0 | 16 | 1 |
| 615 | 1999 | NG | ST-1157 | ST-1157 cc | 0 | 17 | 1 |
| 616 | 1999 | 29E | ST-1419 | ST-1157 cc | 0 | 17 | 1 |
| 617 | 1999 | NG | ST-1421 | ST-1157 cc | 0 | 16 | 1 |
| 618 | 1999 | NG | ST-1421 | ST-1157 cc | 0 | 16 | 1 |
| 619 | 1999 | NG | ST-1649 | ST-1157 cc | 0 | 17 | 1 |
| 620 | 1999 | B | ST-1669 | ST-1157 cc | 0 | 17 | 1 |
| 621 | 1999 | 29E | ST-2404 | ST-1157 cc | 0 | 16 | 1 |
| 622 | 1999 | 29E | ST-3062 | ST-1157 cc | 0 | 17 | 1 |
| 623 | 1999 | NG | ST-3148 | ST-1157 cc | 0 | 16 | 1 |
| 624 | 1999 | NG | ST-3203 | ST-1157 cc | 0 | 17 | 1 |
| 625 | 1999 | 29E | ST-963 | ST-nd cc | 0 | 17 | 0 |
| 626 | 1999 | 29E | ST-963 | ST-nd cc | 0 | 18 | 0 |
| 627 | 1999 | NG | ST-963 | ST-nd cc | 0 | 18 | 0 |
| 628 | 1999 | NG | ST-963 | ST-nd cc | 0 | 18 | 0 |
| 629 | 1999 | NG | ST-963 | ST-nd cc | 0 | 18 | 0 |
| 630 | 1999 | Y | ST-963 | ST-nd cc | 0 | 17 | 0 |
| 631 | 1999 | NG | ST-1117 | ST-nd cc | 0 | 16 | 0 |
| 632 | 1999 | NG | ST-1136 | ST-nd cc | 0 | 16 | 0 |
| 633 | 1999 | NG | ST-1428 | ST-nd cc | 0 | 19 | 0 |
| 634 | 1999 | NG | ST-1431 | ST-nd cc | 0 | 17 | 0 |
| 635 | 1999 | NG | ST-1431 | ST-nd cc | 0 | 17 | 0 |
| 636 | 1999 | B | ST-1444 | ST-nd cc | 0 | 16 | 0 |
| 637 | 1999 | 29E | ST-1630 | ST-nd cc | 0 | 17 | 0 |
| 638 | 1999 | NG | ST-1630 | ST-nd cc | 0 | 16 | 0 |
| 639 | 1999 | Y | ST-1633 | ST-nd cc | 0 | 16 | 0 |
| 640 | 1999 | 29E | ST-1634 | ST-nd cc | 0 | 16 | 0 |
| 641 | 1999 | Y | ST-1635 | ST-nd cc | 0 | 17 | 0 |
| 642 | 1999 | B | ST-1639 | ST-nd cc | 0 | 17 | 0 |
| 643 | 1999 | NG | ST-1646 | ST-nd cc | 0 | 17 | 0 |
| 644 | 1999 | NG | ST-1648 | ST-nd cc | 0 | 17 | 0 |
| 645 | 1999 | B | ST-1653 | ST-nd cc | 0 | 17 | 0 |
| 646 | 1999 | B | ST-1653 | ST-nd cc | 0 | 17 | 0 |
| 647 | 1999 | NG | ST-1668 | ST-nd cc | 0 | 17 | 0 |
| 648 | 1999 | 29E | ST-1678 | ST-nd cc | 0 | 17 | 0 |
| 649 | 1999 | NG | ST-2392 | ST-nd cc | 0 | 17 | 0 |
| 650 | 1999 | NG | ST-2407 | ST-nd cc | 0 | 17 | 0 |
| 651 | 1999 | NG | ST-2426 | ST-nd cc | 0 | 17 | 0 |
| 652 | 1999 | NG | ST-2431 | ST-nd cc | 0 | 16 | 0 |
| 653 | 1999 | NG | ST-2442 | ST-nd cc | 0 | 16 | 0 |
| 654 | 1999 | NG | ST-2447 | ST-nd cc | 0 | 18 | 0 |
| 655 | 1999 | NG | ST-2460 | ST-nd cc | 0 | 16 | 0 |
| 656 | 1999 | W135 | ST-2473 | ST-nd cc | 0 | 16 | 0 |
| 657 | 1999 | NG | ST-2896 | ST-nd cc | 0 | 17 | 0 |
| 658 | 1999 | NG | ST-3112 | ST-nd cc | 0 | 17 | 0 |
| 659 | 1999 | W135 | ST-3170 | ST-nd cc | 0 | 17 | 0 |
| 660 | 1999 | NG | ST-3180 | ST-nd cc | 0 | 16 | 0 |
| 661 | 1999 | NG | ST-3212 | ST-nd cc | 0 | 17 | 0 |
| 662 | 1999 | NG | ST-3225 | ST-nd cc | 0 | 16 | 0 |
| 663 | 1999 | NG | ST-3260 | ST-nd cc | 0 | 16 | 0 |
| 664 | 1999 | 29E | ST-178 | ST-nd cc | 0 | 17 | 1 |
| 665 | 1999 | 29E | ST-212 | ST-nd cc | 0 | 18 | 1 |
| 666 | 1999 | NG | ST-212 | ST-nd cc | 0 | 17 | 1 |
| 667 | 1999 | NG | ST-212 | ST-nd cc | 0 | 17 | 1 |
| 668 | 1999 | NG | ST-212 | ST-nd cc | 0 | 18 | 1 |
| 669 | 1999 | B | ST-1052 | ST-nd cc | 0 | 17 | 1 |
| 670 | 1999 | NG | ST-1117 | ST-nd cc | 0 | 16 | 1 |
| 671 | 1999 | NG | ST-1420 | ST-nd cc | 0 | 17 | 1 |
| 672 | 1999 | NG | ST-1422 | ST-nd cc | 0 | 17 | 1 |
| 673 | 1999 | NG | ST-1434 | ST-nd cc | 0 | 18 | 0 |
| 674 | 1999 | C | ST-1435 | ST-nd cc | 0 | 17 | 1 |
| 675 | 1999 | B | ST-1442 | ST-nd cc | 0 | 17 | 1 |
| 676 | 1999 | NG | ST-1446 | ST-nd cc | 0 | 18 | 1 |
| 677 | 1999 | B | ST-1450 | ST-nd cc | 0 | 17 | 1 |
| 678 | 1999 | C | ST-1451 | ST-nd cc | 0 | 16 | 1 |
| 679 | 1999 | NG | ST-1456 | ST-nd cc | 0 | 16 | 1 |
| 680 | 1999 | NG | ST-1457 | ST-nd cc | 0 | 17 | 1 |
| 681 | 1999 | NG | ST-1459 | ST-nd cc | 0 | 17 | 1 |
| 682 | 1999 | B | ST-1632 | ST-nd cc | 0 | 16 | 1 |
| 683 | 1999 | B | ST-1642 | ST-nd cc | 0 | 17 | 1 |
| 684 | 1999 | NG | ST-1665 | ST-nd cc | 0 | 17 | 1 |
| 685 | 1999 | B | ST-1671 | ST-nd cc | 0 | 17 | 1 |
| 686 | 1999 | B | ST-1672 | ST-nd cc | 0 | 16 | 1 |
| 687 | 1999 | B | ST-1802 | ST-nd cc | 0 | 18 | 1 |
| 688 | 1999 | B | ST-1802 | ST-nd cc | 0 | 17 | 1 |
| 689 | 1999 | NG | ST-1958 | ST-nd cc | 0 | 18 | 1 |
| 690 | 1999 | B | ST-2357 | ST-nd cc | 0 | 16 | 1 |
| 691 | 1999 | NG | ST-2365 | ST-nd cc | 0 | 17 | 1 |
| 692 | 1999 | NG | ST-2392 | ST-nd cc | 0 | 16 | 1 |
| 693 | 1999 | NG | ST-2393 | ST-nd cc | 0 | 16 | 1 |
| 694 | 1999 | NG | ST-2393 | ST-nd cc | 0 | 19 | 1 |
| 695 | 1999 | NG | ST-2432 | ST-nd cc | 0 | 17 | 1 |
| 696 | 1999 | NG | ST-2457 | ST-nd cc | 0 | 17 | 1 |
| 697 | 1999 | B | ST-3063 | ST-nd cc | 0 | 17 | 1 |
| 698 | 1999 | B | ST-3071 | ST-nd cc | 0 | 17 | 1 |
| 699 | 1999 | B | ST-3102 | ST-nd cc | 0 | 17 | 1 |
| 700 | 1999 | B | ST-3157 | ST-nd cc | 0 | 17 | 1 |
| 701 | 1999 | NG | ST-3167 | ST-nd cc | 0 | 17 | 1 |
| 702 | 1999 | B | ST-3269 | ST-nd cc | 0 | 17 | 1 |
| 703 | 1999 | NG | ST-4147 | ST-nd cc | 0 | 16 | 1 |
| 704 | 2001 | C | ST-8 | ST-8 cc | 1 | 35 | 1 |
| 705 | 2001 | B | ST-153 | ST-8 cc | 1 | 39 | 1 |
| 706 | 2001 | W135 | ST-11 | ST-11 cc | 1 | 33 | 0 |
| 707 | 2001 | W135 | ST-11 | ST-11 cc | 1 | 47 | 0 |
| 708 | 2001 | C | ST-11 | ST-11 cc | 1 | 56 | 1 |
| 709 | 2001 | C | ST-11 | ST-11 cc | 1 | 37 | 1 |
| 710 | 2001 | C | ST-11 | ST-11 cc | 1 | 28 | 1 |
| 711 | 2001 | C | ST-11 | ST-11 cc | 1 | 39 | 1 |
| 712 | 2001 | C | ST-11 | ST-11 cc | 1 | 54 | 1 |
| 713 | 2001 | C | ST-11 | ST-11 cc | 1 | 60 | 1 |
| 714 | 2001 | C | ST-11 | ST-11 cc | 1 | 21 | 1 |
| 715 | 2001 | C | ST-11 | ST-11 cc | 1 | 27 | 1 |
| 716 | 2001 | C | ST-11 | ST-11 cc | 1 | 25 | 1 |
| 717 | 2001 | C | ST-11 | ST-11 cc | 1 | 43 | 1 |
| 718 | 2001 | C | ST-11 | ST-11 cc | 1 | 82 | 1 |
| 719 | 2001 | C | ST-11 | ST-11 cc | 1 | 28 | 1 |
| 720 | 2001 | C | ST-11 | ST-11 cc | 1 | 40 | 1 |
| 721 | 2001 | C | ST-11 | ST-11 cc | 1 | 2 | 1 |
| 722 | 2001 | C | ST-11 | ST-11 cc | 1 | 28 | 1 |
| 723 | 2001 | C | ST-11 | ST-11 cc | 1 | 12 | 1 |
| 724 | 2001 | C | ST-11 | ST-11 cc | 1 | <1 | 1 |
| 725 | 2001 | C | ST-11 | ST-11 cc | 1 | 46 | 1 |
| 726 | 2001 | C | ST-11 | ST-11 cc | 1 | 41 | 1 |
| 727 | 2001 | C | ST-11 | ST-11 cc | 1 | 73 | 1 |
| 728 | 2001 | C | ST-11 | ST-11 cc | 1 | 46 | 1 |
| 729 | 2001 | C | ST-11 | ST-11 cc | 1 | 37 | 1 |
| 730 | 2001 | C | ST-11 | ST-11 cc | 1 | 23 | 1 |
| 731 | 2001 | C | ST-11 | ST-11 cc | 1 | 21 | 1 |
| 732 | 2001 | C | ST-11 | ST-11 cc | 1 | 21 | 1 |
| 733 | 2001 | C | ST-11 | ST-11 cc | 1 | 88 | 1 |
| 734 | 2001 | B | ST-11 | ST-11 cc | 1 | 17 | 1 |
| 735 | 2001 | W135 | ST-11 | ST-11 cc | 1 | 28 | 1 |
| 736 | 2001 | W135 | ST-11 | ST-11 cc | 1 | 34 | 1 |
| 737 | 2001 | W135 | ST-11 | ST-11 cc | 1 | <1 | 1 |
| 738 | 2001 | W135 | ST-11 | ST-11 cc | 1 | <1 | 1 |
| 739 | 2001 | C | ST-2994 | ST-11 cc | 1 | 49 | 1 |
| 740 | 2001 | B | ST-18 | ST-18 cc | 1 | 38 | 1 |
| 741 | 2001 | W135 | ST-22 | ST-22 cc | 1 | <1 | 0 |
| 742 | 2001 | W135 | ST-1397 | ST-22 cc | 1 | <1 | 0 |
| 743 | 2001 | B | ST-32 | ST-32 cc | 1 | 8 | 0 |
| 744 | 2001 | B | ST-32 | ST-32 cc | 1 | 2 | 1 |
| 745 | 2001 | B | ST-32 | ST-32 cc | 1 | 3 | 1 |
| 746 | 2001 | B | ST-33 | ST-32 cc | 1 | 49 | 1 |
| 747 | 2001 | B | ST-34 | ST-32 cc | 1 | 5 | 1 |
| 748 | 2001 | B | ST-34 | ST-32 cc | 1 | 15 | 1 |
| 749 | 2001 | B | ST-34 | ST-32 cc | 1 | 21 | 1 |
| 750 | 2001 | B | ST-34 | ST-32 cc | 1 | 47 | 1 |
| 751 | 2001 | B | ST-74 | ST-32 cc | 1 | 57 | 1 |
| 752 | 2001 | B | ST-1398 | ST-32 cc | 1 | 2 | 1 |
| 753 | 2001 | B | ST-1784 | ST-32 cc | 1 | 10 | 1 |
| 754 | 2001 | B | ST-35 | ST-35 cc | 1 | 1 | 1 |
| 755 | 2001 | B | ST-457 | ST-35 cc | 1 | <1 | 1 |
| 756 | 2001 | B | ST-40 | ST-41/44 cc | 1 | 18 | 1 |
| 757 | 2001 | C | ST-41 | ST-41/44 cc | 1 | 39 | 1 |
| 758 | 2001 | B | ST-41 | ST-41/44 cc | 1 | 1 | 1 |
| 759 | 2001 | B | ST-41 | ST-41/44 cc | 1 | 1 | 1 |
| 760 | 2001 | B | ST-41 | ST-41/44 cc | 1 | 2 | 1 |
| 761 | 2001 | B | ST-41 | ST-41/44 cc | 1 | 3 | 1 |
| 762 | 2001 | B | ST-41 | ST-41/44 cc | 1 | 3 | 1 |
| 763 | 2001 | B | ST-41 | ST-41/44 cc | 1 | 3 | 1 |
| 764 | 2001 | B | ST-41 | ST-41/44 cc | 1 | 6 | 1 |
| 765 | 2001 | B | ST-41 | ST-41/44 cc | 1 | 7 | 1 |
| 766 | 2001 | B | ST-41 | ST-41/44 cc | 1 | 8 | 1 |
| 767 | 2001 | B | ST-41 | ST-41/44 cc | 1 | 9 | 1 |
| 768 | 2001 | B | ST-41 | ST-41/44 cc | 1 | 12 | 1 |
| 769 | 2001 | B | ST-41 | ST-41/44 cc | 1 | 13 | 1 |
| 770 | 2001 | B | ST-41 | ST-41/44 cc | 1 | 17 | 1 |
| 771 | 2001 | B | ST-41 | ST-41/44 cc | 1 | 17 | 1 |
| 772 | 2001 | B | ST-41 | ST-41/44 cc | 1 | 17 | 1 |
| 773 | 2001 | B | ST-41 | ST-41/44 cc | 1 | 22 | 1 |
| 774 | 2001 | B | ST-41 | ST-41/44 cc | 1 | 24 | 1 |
| 775 | 2001 | B | ST-41 | ST-41/44 cc | 1 | 27 | 1 |
| 776 | 2001 | B | ST-41 | ST-41/44 cc | 1 | 29 | 1 |
| 777 | 2001 | B | ST-41 | ST-41/44 cc | 1 | 49 | 1 |
| 778 | 2001 | B | ST-41 | ST-41/44 cc | 1 | <1 | 1 |
| 779 | 2001 | B | ST-41 | ST-41/44 cc | 1 | <1 | 1 |
| 780 | 2001 | B | ST-41 | ST-41/44 cc | 1 | <1 | 1 |
| 781 | 2001 | B | ST-43 | ST-41/44 cc | 1 | 1 | 1 |
| 782 | 2001 | B | ST-154 | ST-41/44 cc | 1 | 20 | 1 |
| 783 | 2001 | B | ST-154 | ST-41/44 cc | 1 | 33 | 1 |
| 784 | 2001 | B | ST-207 | ST-41/44 cc | 1 | 7 | 1 |
| 785 | 2001 | B | ST-274 | ST-41/44 cc | 1 | 23 | 1 |
| 786 | 2001 | B | ST-318 | ST-41/44 cc | 1 | 41 | 1 |
| 787 | 2001 | B | ST-340 | ST-41/44 cc | 1 | 1 | 1 |
| 788 | 2001 | B | ST-340 | ST-41/44 cc | 1 | 4 | 1 |
| 789 | 2001 | B | ST-340 | ST-41/44 cc | 1 | 58 | 1 |
| 790 | 2001 | B | ST-1097 | ST-41/44 cc | 1 | 19 | 1 |
| 791 | 2001 | B | ST-1194 | ST-41/44 cc | 1 | 16 | 1 |
| 792 | 2001 | B | ST-1915 | ST-41/44 cc | 1 | 5 | 1 |
| 793 | 2001 | B | ST-3669 | ST-41/44 cc | 1 | <1 | 1 |
| 794 | 2001 | B | ST-3794 | ST-41/44 cc | 1 | <1 | 1 |
| 795 | 2001 | B | ST-4431 | ST-41/44 cc | 1 | 10 | 1 |
| 796 | 2001 | B | ST-4840 | ST-41/44 cc | 1 | <1 | 1 |
| 797 | 2001 | 29E | ST-60 | ST-60 cc | 1 | 20 | 0 |
| 798 | 2001 | B | ST-60 | ST-60 cc | 1 | <1 | 0 |
| 799 | 2001 | B | ST-103 | ST-103 cc | 1 | <1 | 0 |
| 800 | 2001 | Y | ST-167 | ST-167 cc | 1 | 55 | 1 |
| 801 | 2001 | B | ST-213 | ST-213 cc | 1 | 1 | 0 |
| 802 | 2001 | B | ST-213 | ST-213 cc | 1 | 16 | 0 |
| 803 | 2001 | B | ST-213 | ST-213 cc | 1 | <1 | 1 |
| 804 | 2001 | B | ST-1990 | ST-269 cc | 1 | 0 | 0 |
| 805 | 2001 | C | ST-269 | ST-269 cc | 1 | 21 | 1 |
| 806 | 2001 | B | ST-269 | ST-269 cc | 1 | 0 | 1 |
| 807 | 2001 | B | ST-269 | ST-269 cc | 1 | 1 | 1 |
| 808 | 2001 | B | ST-269 | ST-269 cc | 1 | 2 | 1 |
| 809 | 2001 | B | ST-269 | ST-269 cc | 1 | 2 | 1 |
| 810 | 2001 | B | ST-269 | ST-269 cc | 1 | 3 | 1 |
| 811 | 2001 | B | ST-269 | ST-269 cc | 1 | 3 | 1 |
| 812 | 2001 | B | ST-269 | ST-269 cc | 1 | 11 | 1 |
| 813 | 2001 | B | ST-269 | ST-269 cc | 1 | 13 | 1 |
| 814 | 2001 | B | ST-269 | ST-269 cc | 1 | 16 | 1 |
| 815 | 2001 | B | ST-269 | ST-269 cc | 1 | 17 | 1 |
| 816 | 2001 | B | ST-269 | ST-269 cc | 1 | 18 | 1 |
| 817 | 2001 | B | ST-269 | ST-269 cc | 1 | 19 | 1 |
| 818 | 2001 | B | ST-269 | ST-269 cc | 1 | 43 | 1 |
| 819 | 2001 | B | ST-269 | ST-269 cc | 1 | 57 | 1 |
| 820 | 2001 | B | ST-269 | ST-269 cc | 1 | 73 | 1 |
| 821 | 2001 | B | ST-269 | ST-269 cc | 1 | <1 | 1 |
| 822 | 2001 | B | ST-269 | ST-269 cc | 1 | <1 | 1 |
| 823 | 2001 | B | ST-275 | ST-269 cc | 1 | 1 | 1 |
| 824 | 2001 | B | ST-275 | ST-269 cc | 1 | 8 | 1 |
| 825 | 2001 | B | ST-275 | ST-269 cc | 1 | 19 | 1 |
| 826 | 2001 | B | ST-275 | ST-269 cc | 1 | <1 | 1 |
| 827 | 2001 | B | ST-283 | ST-269 cc | 1 | <1 | 1 |
| 828 | 2001 | B | ST-479 | ST-269 cc | 1 | 2 | 1 |
| 829 | 2001 | B | ST-1163 | ST-269 cc | 1 | 17 | 1 |
| 830 | 2001 | B | ST-1195 | ST-269 cc | 1 | 4 | 1 |
| 831 | 2001 | B | ST-1195 | ST-269 cc | 1 | 38 | 1 |
| 832 | 2001 | B | ST-1214 | ST-269 cc | 1 | 19 | 1 |
| 833 | 2001 | B | ST-1214 | ST-269 cc | 1 | 19 | 1 |
| 834 | 2001 | B | ST-1395 | ST-269 cc | 1 | 4 | 1 |
| 835 | 2001 | B | ST-1942 | ST-269 cc | 1 | 19 | 1 |
| 836 | 2001 | B | ST-3216 | ST-364 cc | 1 | <1 | 1 |
| 837 | 2001 | B | ST-461 | ST-461 cc | 1 | 2 | 0 |
| 838 | 2001 | B | ST-1157 | ST-1157 cc | 1 | 5 | 0 |
| 839 | 2001 | X | ST-5338 | ST-nd cc | 1 | 1 | 0 |
| 840 | 2001 | B | ST-5340 | ST-nd cc | 1 | <1 | 0 |
| 841 | 2001 | B | ST-5349 | ST-nd cc | 1 | <1 | 0 |
| 842 | 2001 | NG | ST-5349 | ST-nd cc | 1 | <1 | 0 |
| 843 | 2001 | B | ST-5497 | ST-nd cc | 1 | 42 | 0 |
| 844 | 2001 | B | ST-271 | ST-nd cc | 1 | 8 | 1 |
| 845 | 2001 | B | ST-282 | ST-nd cc | 1 | 1 | 1 |
| 846 | 2001 | B | ST-286 | ST-nd cc | 1 | <1 | 1 |
| 847 | 2001 | B | ST-1575 | ST-nd cc | 1 | 1 | 1 |
| 848 | 2001 | B | ST-1935 | ST-nd cc | 1 | 13 | 1 |
| 849 | 2001 | B | ST-3475 | ST-nd cc | 1 | 28 | 1 |
| 850 | 2001 | B | ST-5160 | ST-nd cc | 1 | 80 | 1 |
| 851 | 2001 | B | ST-5282 | ST-nd cc | 1 | <1 | 1 |
| 852 | 2001 | B | ST-5283 | ST-nd cc | 1 | 0 | 1 |
| 853 | 2001 | B | ST-5287 | ST-nd cc | 1 | 6 | 1 |
| 854 | 2001 | B | ST-5328 | ST-nd cc | 1 | 4 | 1 |
| 855 | 2001 | B | ST-5331 | ST-nd cc | 1 | 36 | 1 |
| 856 | 2001 | C | ST-5351 | ST-nd cc | 1 | 18 | 1 |
| 857 | 2001 | B | ST-5486 | ST-nd cc | 1 | 19 | 1 |
| 858 | 2001 | C | ST-8 | ST-8 cc | 0 | 17 | 0 |
| 859 | 2001 | B | ST-153 | ST-8 cc | 0 | 16 | 1 |
| 860 | 2001 | W135 | ST-11 | ST-11 cc | 0 | 19 | 1 |
| 861 | 2001 | NG | ST-22 | ST-22 cc | 0 | 16 | 0 |
| 862 | 2001 | NG | ST-22 | ST-22 cc | 0 | 17 | 0 |
| 863 | 2001 | NG | ST-22 | ST-22 cc | 0 | 19 | 0 |
| 864 | 2001 | NG | ST-22 | ST-22 cc | 0 | 17 | 0 |
| 865 | 2001 | NG | ST-22 | ST-22 cc | 0 | 16 | 0 |
| 866 | 2001 | W135 | ST-22 | ST-22 cc | 0 | 17 | 0 |
| 867 | 2001 | W135 | ST-22 | ST-22 cc | 0 | 16 | 0 |
| 868 | 2001 | W135 | ST-22 | ST-22 cc | 0 | 16 | 0 |
| 869 | 2001 | W135 | ST-22 | ST-22 cc | 0 | 18 | 0 |
| 870 | 2001 | NG | ST-184 | ST-22 cc | 0 | 17 | 0 |
| 871 | 2001 | NG | ST-184 | ST-22 cc | 0 | 17 | 0 |
| 872 | 2001 | NG | ST-184 | ST-22 cc | 0 | 17 | 0 |
| 873 | 2001 | NG | ST-184 | ST-22 cc | 0 | 17 | 0 |
| 874 | 2001 | NG | ST-184 | ST-22 cc | 0 | 17 | 0 |
| 875 | 2001 | NG | ST-184 | ST-22 cc | 0 | 17 | 0 |
| 876 | 2001 | NG | ST-184 | ST-22 cc | 0 | 18 | 0 |
| 877 | 2001 | W135 | ST-184 | ST-22 cc | 0 | 16 | 0 |
| 878 | 2001 | W135 | ST-184 | ST-22 cc | 0 | 16 | 0 |
| 879 | 2001 | W135 | ST-184 | ST-22 cc | 0 | 18 | 0 |
| 880 | 2001 | W135 | ST-184 | ST-22 cc | 0 | 17 | 0 |
| 881 | 2001 | W135 | ST-184 | ST-22 cc | 0 | 17 | 0 |
| 882 | 2001 | W135 | ST-184 | ST-22 cc | 0 | 18 | 0 |
| 883 | 2001 | W135 | ST-184 | ST-22 cc | 0 | 16 | 0 |
| 884 | 2001 | NG | ST-903 | ST-22 cc | 0 | 17 | 0 |
| 885 | 2001 | W135 | ST-1158 | ST-22 cc | 0 | 16 | 0 |
| 886 | 2001 | W135 | ST-1221 | ST-22 cc | 0 | 16 | 0 |
| 887 | 2001 | NG | ST-1224 | ST-22 cc | 0 | 16 | 0 |
| 888 | 2001 | W135 | ST-1224 | ST-22 cc | 0 | 17 | 0 |
| 889 | 2001 | NG | ST-1476 | ST-22 cc | 0 | 17 | 0 |
| 890 | 2001 | W135 | ST-1476 | ST-22 cc | 0 | 17 | 0 |
| 891 | 2001 | W135 | ST-1476 | ST-22 cc | 0 | 17 | 0 |
| 892 | 2001 | NG | ST-1617 | ST-22 cc | 0 | 16 | 0 |
| 893 | 2001 | NG | ST-1617 | ST-22 cc | 0 | 16 | 0 |
| 894 | 2001 | NG | ST-1617 | ST-22 cc | 0 | 16 | 0 |
| 895 | 2001 | W135 | ST-1617 | ST-22 cc | 0 | 16 | 0 |
| 896 | 2001 | W135 | ST-1617 | ST-22 cc | 0 | 17 | 0 |
| 897 | 2001 | W135 | ST-1617 | ST-22 cc | 0 | 18 | 0 |
| 898 | 2001 | W135 | ST-1659 | ST-22 cc | 0 | 16 | 0 |
| 899 | 2001 | W135 | ST-1659 | ST-22 cc | 0 | 17 | 0 |
| 900 | 2001 | NG | ST-1667 | ST-22 cc | 0 | 16 | 0 |
| 901 | 2001 | NG | ST-1667 | ST-22 cc | 0 | 16 | 0 |
| 902 | 2001 | W135 | ST-1667 | ST-22 cc | 0 | 17 | 0 |
| 903 | 2001 | W135 | ST-1766 | ST-22 cc | 0 | 16 | 0 |
| 904 | 2001 | NG | ST-2180 | ST-22 cc | 0 | 16 | 0 |
| 905 | 2001 | NG | ST-2180 | ST-22 cc | 0 | 16 | 0 |
| 906 | 2001 | W135 | ST-2639 | ST-22 cc | 0 | 18 | 0 |
| 907 | 2001 | NG | ST-3172 | ST-22 cc | 0 | 17 | 0 |
| 908 | 2001 | W135 | ST-3195 | ST-22 cc | 0 | 17 | 0 |
| 909 | 2001 | NG | ST-184 | ST-22 cc | 0 | 17 | 1 |
| 910 | 2001 | W135 | ST-1224 | ST-22 cc | 0 | 16 | 1 |
| 911 | 2001 | NG | ST-1264 | ST-22 cc | 0 | 17 | 1 |
| 912 | 2001 | W135 | ST-2642 | ST-22 cc | 0 | 16 | 1 |
| 913 | 2001 | NG | ST-23 | ST-23 cc | 0 | 16 | 0 |
| 914 | 2001 | NG | ST-23 | ST-23 cc | 0 | 17 | 0 |
| 915 | 2001 | NG | ST-23 | ST-23 cc | 0 | 17 | 0 |
| 916 | 2001 | NG | ST-23 | ST-23 cc | 0 | 17 | 0 |
| 917 | 2001 | NG | ST-23 | ST-23 cc | 0 | 16 | 0 |
| 918 | 2001 | NG | ST-23 | ST-23 cc | 0 | 17 | 0 |
| 919 | 2001 | W135 | ST-23 | ST-23 cc | 0 | 16 | 0 |
| 920 | 2001 | NG | ST-2419 | ST-23 cc | 0 | 16 | 0 |
| 921 | 2001 | NG | ST-23 | ST-23 cc | 0 | 17 | 1 |
| 922 | 2001 | NG | ST-23 | ST-23 cc | 0 | 17 | 1 |
| 923 | 2001 | NG | ST-23 | ST-23 cc | 0 | 17 | 1 |
| 924 | 2001 | NG | ST-23 | ST-23 cc | 0 | 17 | 1 |
| 925 | 2001 | NG | ST-23 | ST-23 cc | 0 | 16 | 1 |
| 926 | 2001 | NG | ST-23 | ST-23 cc | 0 | 17 | 1 |
| 927 | 2001 | NG | ST-23 | ST-23 cc | 0 | 16 | 1 |
| 928 | 2001 | NG | ST-1625 | ST-23 cc | 0 | 17 | 1 |
| 929 | 2001 | Y | ST-2421 | ST-23 cc | 0 | 16 | 1 |
| 930 | 2001 | NG | ST-32 | ST-32 cc | 0 | 17 | 1 |
| 931 | 2001 | NG | ST-32 | ST-32 cc | 0 | 16 | 1 |
| 932 | 2001 | NG | ST-34 | ST-32 cc | 0 | 16 | 1 |
| 933 | 2001 | B | ST-74 | ST-32 cc | 0 | 17 | 1 |
| 934 | 2001 | B | ST-264 | ST-32 cc | 0 | 17 | 1 |
| 935 | 2001 | B | ST-2017 | ST-32 cc | 0 | 17 | 1 |
| 936 | 2001 | NG | ST-35 | ST-35 cc | 0 | 17 | 0 |
| 937 | 2001 | NG | ST-35 | ST-35 cc | 0 | 16 | 1 |
| 938 | 2001 | B | ST-457 | ST-35 cc | 0 | 17 | 1 |
| 939 | 2001 | B | ST-457 | ST-35 cc | 0 | 17 | 1 |
| 940 | 2001 | B | ST-457 | ST-35 cc | 0 | 17 | 1 |
| 941 | 2001 | NG | ST-457 | ST-35 cc | 0 | 18 | 1 |
| 942 | 2001 | NG | ST-809 | ST-35 cc | 0 | 16 | 1 |
| 943 | 2001 | NG | ST-809 | ST-35 cc | 0 | 17 | 1 |
| 944 | 2001 | NG | ST-1679 | ST-35 cc | 0 | 18 | 1 |
| 945 | 2001 | NG | ST-2374 | ST-35 cc | 0 | 17 | 1 |
| 946 | 2001 | B | ST-2380 | ST-35 cc | 0 | 16 | 1 |
| 947 | 2001 | B | ST-2437 | ST-35 cc | 0 | 16 | 1 |
| 948 | 2001 | NG | ST-2437 | ST-35 cc | 0 | 16 | 1 |
| 949 | 2001 | B | ST-2574 | ST-35 cc | 0 | 16 | 1 |
| 950 | 2001 | B | ST-2574 | ST-35 cc | 0 | 17 | 1 |
| 951 | 2001 | B | ST-3076 | ST-35 cc | 0 | 18 | 1 |
| 952 | 2001 | B | ST-41 | ST-41/44 cc | 0 | 18 | 1 |
| 953 | 2001 | B | ST-41 | ST-41/44 cc | 0 | 16 | 1 |
| 954 | 2001 | B | ST-41 | ST-41/44 cc | 0 | 17 | 1 |
| 955 | 2001 | NG | ST-41 | ST-41/44 cc | 0 | 17 | 1 |
| 956 | 2001 | NG | ST-41 | ST-41/44 cc | 0 | 17 | 1 |
| 957 | 2001 | NG | ST-41 | ST-41/44 cc | 0 | 16 | 1 |
| 958 | 2001 | NG | ST-41 | ST-41/44 cc | 0 | 17 | 1 |
| 959 | 2001 | NG | ST-41 | ST-41/44 cc | 0 | 16 | 1 |
| 960 | 2001 | NG | ST-41 | ST-41/44 cc | 0 | 14 | 1 |
| 961 | 2001 | NG | ST-41 | ST-41/44 cc | 0 | 18 | 1 |
| 962 | 2001 | B | ST-43 | ST-41/44 cc | 0 | 17 | 1 |
| 963 | 2001 | B | ST-43 | ST-41/44 cc | 0 | 16 | 1 |
| 964 | 2001 | B | ST-43 | ST-41/44 cc | 0 | 16 | 1 |
| 965 | 2001 | B | ST-43 | ST-41/44 cc | 0 | 16 | 1 |
| 966 | 2001 | NG | ST-44 | ST-41/44 cc | 0 | 17 | 1 |
| 967 | 2001 | B | ST-136 | ST-41/44 cc | 0 | 17 | 1 |
| 968 | 2001 | NG | ST-154 | ST-41/44 cc | 0 | 17 | 1 |
| 969 | 2001 | NG | ST-180 | ST-41/44 cc | 0 | 17 | 1 |
| 970 | 2001 | NG | ST-180 | ST-41/44 cc | 0 | 17 | 1 |
| 971 | 2001 | NG | ST-206 | ST-41/44 cc | 0 | 17 | 1 |
| 972 | 2001 | NG | ST-409 | ST-41/44 cc | 0 | 16 | 1 |
| 973 | 2001 | B | ST-437 | ST-41/44 cc | 0 | 16 | 1 |
| 974 | 2001 | NG | ST-482 | ST-41/44 cc | 0 | 16 | 1 |
| 975 | 2001 | B | ST-1097 | ST-41/44 cc | 0 | 16 | 1 |
| 976 | 2001 | B | ST-1097 | ST-41/44 cc | 0 | 17 | 1 |
| 977 | 2001 | B | ST-1097 | ST-41/44 cc | 0 | 18 | 1 |
| 978 | 2001 | NG | ST-1097 | ST-41/44 cc | 0 | 16 | 1 |
| 979 | 2001 | NG | ST-1228 | ST-41/44 cc | 0 | 16 | 1 |
| 980 | 2001 | B | ST-1399 | ST-41/44 cc | 0 | 17 | 1 |
| 981 | 2001 | B | ST-1414 | ST-41/44 cc | 0 | 16 | 1 |
| 982 | 2001 | B | ST-1415 | ST-41/44 cc | 0 | 17 | 1 |
| 983 | 2001 | NG | ST-1857 | ST-41/44 cc | 0 | 17 | 1 |
| 984 | 2001 | NG | ST-1957 | ST-41/44 cc | 0 | 16 | 1 |
| 985 | 2001 | B | ST-2080 | ST-41/44 cc | 0 | 17 | 1 |
| 986 | 2001 | B | ST-2189 | ST-41/44 cc | 0 | 16 | 1 |
| 987 | 2001 | B | ST-2364 | ST-41/44 cc | 0 | 17 | 1 |
| 988 | 2001 | NG | ST-2413 | ST-41/44 cc | 0 | 16 | 1 |
| 989 | 2001 | NG | ST-2413 | ST-41/44 cc | 0 | 18 | 1 |
| 990 | 2001 | NG | ST-2413 | ST-41/44 cc | 0 | 16 | 1 |
| 991 | 2001 | B | ST-2417 | ST-41/44 cc | 0 | 16 | 1 |
| 992 | 2001 | B | ST-2631 | ST-41/44 cc | 0 | 17 | 1 |
| 993 | 2001 | B | ST-2644 | ST-41/44 cc | 0 | 16 | 1 |
| 994 | 2001 | B | ST-3048 | ST-41/44 cc | 0 | 17 | 1 |
| 995 | 2001 | B | ST-3049 | ST-41/44 cc | 0 | 17 | 1 |
| 996 | 2001 | NG | ST-3248 | ST-41/44 cc | 0 | 17 | 1 |
| 997 | 2001 | NG | ST-3259 | ST-41/44 cc | 0 | 18 | 1 |
| 998 | 2001 | B | ST-4791 | ST-41/44 cc | 0 | 17 | 1 |
| 999 | 2001 | NG | ST-53 | ST-53 cc | 0 | 16 | 0 |
| 1000 | 2001 | NG | ST-53 | ST-53 cc | 0 | 16 | 0 |
| 1001 | 2001 | NG | ST-53 | ST-53 cc | 0 | 16 | 0 |
| 1002 | 2001 | NG | ST-53 | ST-53 cc | 0 | 17 | 0 |
| 1003 | 2001 | NG | ST-53 | ST-53 cc | 0 | 16 | 0 |
| 1004 | 2001 | NG | ST-53 | ST-53 cc | 0 | 17 | 0 |
| 1005 | 2001 | NG | ST-53 | ST-53 cc | 0 | 18 | 0 |
| 1006 | 2001 | NG | ST-53 | ST-53 cc | 0 | 16 | 0 |
| 1007 | 2001 | NG | ST-53 | ST-53 cc | 0 | 18 | 0 |
| 1008 | 2001 | NG | ST-53 | ST-53 cc | 0 | 17 | 0 |
| 1009 | 2001 | NG | ST-53 | ST-53 cc | 0 | 17 | 0 |
| 1010 | 2001 | NG | ST-53 | ST-53 cc | 0 | 16 | 0 |
| 1011 | 2001 | NG | ST-53 | ST-53 cc | 0 | 17 | 0 |
| 1012 | 2001 | NG | ST-53 | ST-53 cc | 0 | 17 | 0 |
| 1013 | 2001 | NG | ST-53 | ST-53 cc | 0 | 17 | 0 |
| 1014 | 2001 | NG | ST-53 | ST-53 cc | 0 | 16 | 0 |
| 1015 | 2001 | NG | ST-53 | ST-53 cc | 0 | 17 | 0 |
| 1016 | 2001 | NG | ST-53 | ST-53 cc | 0 | 19 | 0 |
| 1017 | 2001 | NG | ST-53 | ST-53 cc | 0 | 16 | 0 |
| 1018 | 2001 | NG | ST-53 | ST-53 cc | 0 | 16 | 0 |
| 1019 | 2001 | NG | ST-53 | ST-53 cc | 0 | 17 | 0 |
| 1020 | 2001 | NG | ST-53 | ST-53 cc | 0 | 16 | 0 |
| 1021 | 2001 | NG | ST-53 | ST-53 cc | 0 | 17 | 0 |
| 1022 | 2001 | NG | ST-53 | ST-53 cc | 0 | 16 | 0 |
| 1023 | 2001 | NG | ST-53 | ST-53 cc | 0 | 17 | 0 |
| 1024 | 2001 | NG | ST-53 | ST-53 cc | 0 | 18 | 0 |
| 1025 | 2001 | NG | ST-53 | ST-53 cc | 0 | 18 | 0 |
| 1026 | 2001 | NG | ST-53 | ST-53 cc | 0 | 18 | 0 |
| 1027 | 2001 | NG | ST-2069 | ST-53 cc | 0 | 17 | 0 |
| 1028 | 2001 | NG | ST-2069 | ST-53 cc | 0 | 17 | 0 |
| 1029 | 2001 | NG | ST-2069 | ST-53 cc | 0 | 15 | 0 |
| 1030 | 2001 | NG | ST-53 | ST-53 cc | 0 | 16 | 0 |
| 1031 | 2001 | NG | ST-53 | ST-53 cc | 0 | 16 | 0 |
| 1032 | 2001 | NG | ST-53 | ST-53 cc | 0 | 16 | 0 |
| 1033 | 2001 | NG | ST-53 | ST-53 cc | 0 | 17 | 0 |
| 1034 | 2001 | 29E | ST-60 | ST-60 cc | 0 | 16 | 0 |
| 1035 | 2001 | 29E | ST-60 | ST-60 cc | 0 | 16 | 0 |
| 1036 | 2001 | 29E | ST-60 | ST-60 cc | 0 | 17 | 0 |
| 1037 | 2001 | 29E | ST-60 | ST-60 cc | 0 | 16 | 0 |
| 1038 | 2001 | B | ST-60 | ST-60 cc | 0 | 17 | 0 |
| 1039 | 2001 | NG | ST-60 | ST-60 cc | 0 | 17 | 0 |
| 1040 | 2001 | NG | ST-60 | ST-60 cc | 0 | 17 | 0 |
| 1041 | 2001 | NG | ST-60 | ST-60 cc | 0 | 16 | 0 |
| 1042 | 2001 | NG | ST-60 | ST-60 cc | 0 | 16 | 0 |
| 1043 | 2001 | NG | ST-60 | ST-60 cc | 0 | 17 | 0 |
| 1044 | 2001 | NG | ST-60 | ST-60 cc | 0 | 16 | 0 |
| 1045 | 2001 | NG | ST-60 | ST-60 cc | 0 | 16 | 0 |
| 1046 | 2001 | NG | ST-60 | ST-60 cc | 0 | 18 | 0 |
| 1047 | 2001 | NG | ST-60 | ST-60 cc | 0 | 17 | 0 |
| 1048 | 2001 | NG | ST-60 | ST-60 cc | 0 | 19 | 0 |
| 1049 | 2001 | NG | ST-466 | ST-60 cc | 0 | 16 | 0 |
| 1050 | 2001 | 29E | ST-895 | ST-60 cc | 0 | 18 | 0 |
| 1051 | 2001 | 29E | ST-905 | ST-60 cc | 0 | 17 | 0 |
| 1052 | 2001 | 29E | ST-905 | ST-60 cc | 0 | 17 | 0 |
| 1053 | 2001 | 29E | ST-905 | ST-60 cc | 0 | 17 | 0 |
| 1054 | 2001 | 29E | ST-913 | ST-60 cc | 0 | 17 | 0 |
| 1055 | 2001 | NG | ST-913 | ST-60 cc | 0 | 17 | 0 |
| 1056 | 2001 | NG | ST-913 | ST-60 cc | 0 | 16 | 0 |
| 1057 | 2001 | NG | ST-1243 | ST-60 cc | 0 | 16 | 0 |
| 1058 | 2001 | 29E | ST-1383 | ST-60 cc | 0 | 17 | 0 |
| 1059 | 2001 | 29E | ST-1383 | ST-60 cc | 0 | 16 | 0 |
| 1060 | 2001 | NG | ST-1383 | ST-60 cc | 0 | 16 | 0 |
| 1061 | 2001 | 29E | ST-1677 | ST-60 cc | 0 | 16 | 0 |
| 1062 | 2001 | 29E | ST-1754 | ST-60 cc | 0 | 17 | 0 |
| 1063 | 2001 | 29E | ST-1754 | ST-60 cc | 0 | 17 | 0 |
| 1064 | 2001 | 29E | ST-3235 | ST-60 cc | 0 | 17 | 0 |
| 1065 | 2001 | 29E | ST-3235 | ST-60 cc | 0 | 17 | 0 |
| 1066 | 2001 | 29E | ST-60 | ST-60 cc | 0 | 16 | 1 |
| 1067 | 2001 | NG | ST-60 | ST-60 cc | 0 | 16 | 0 |
| 1068 | 2001 | NG | ST-913 | ST-60 cc | 0 | 17 | 1 |
| 1069 | 2001 | 29E | ST-3234 | ST-60 cc | 0 | 17 | 1 |
| 1070 | 2001 | NG | ST-784 | ST-92 cc | 0 | 17 | 0 |
| 1071 | 2001 | NG | ST-103 | ST-103 cc | 0 | 17 | 0 |
| 1072 | 2001 | NG | ST-103 | ST-103 cc | 0 | 17 | 0 |
| 1073 | 2001 | Z | ST-103 | ST-103 cc | 0 | 17 | 0 |
| 1074 | 2001 | NG | ST-862 | ST-103 cc | 0 | 17 | 0 |
| 1075 | 2001 | 29E | ST-2399 | ST-103 cc | 0 | 17 | 0 |
| 1076 | 2001 | Z | ST-2399 | ST-103 cc | 0 | 17 | 0 |
| 1077 | 2001 | NG | ST-3144 | ST-103 cc | 0 | 16 | 0 |
| 1078 | 2001 | Z | ST-103 | ST-103 cc | 0 | 19 | 1 |
| 1079 | 2001 | Z | ST-1878 | ST-103 cc | 0 | 17 | 1 |
| 1080 | 2001 | NG | ST-2006 | ST-103 cc | 0 | 18 | 1 |
| 1081 | 2001 | B | ST-162 | ST-162 cc | 0 | 16 | 1 |
| 1082 | 2001 | B | ST-162 | ST-162 cc | 0 | 17 | 1 |
| 1083 | 2001 | NG | ST-167 | ST-167 cc | 0 | 16 | 0 |
| 1084 | 2001 | NG | ST-167 | ST-167 cc | 0 | 16 | 0 |
| 1085 | 2001 | NG | ST-167 | ST-167 cc | 0 | 16 | 0 |
| 1086 | 2001 | NG | ST-168 | ST-167 cc | 0 | 18 | 0 |
| 1087 | 2001 | NG | ST-279 | ST-167 cc | 0 | 17 | 0 |
| 1088 | 2001 | NG | ST-767 | ST-167 cc | 0 | 17 | 0 |
| 1089 | 2001 | NG | ST-767 | ST-167 cc | 0 | 17 | 0 |
| 1090 | 2001 | NG | ST-884 | ST-167 cc | 0 | 17 | 0 |
| 1091 | 2001 | NG | ST-3166 | ST-167 cc | 0 | 16 | 0 |
| 1092 | 2001 | Y | ST-167 | ST-167 cc | 0 | 17 | 1 |
| 1093 | 2001 | Y | ST-168 | ST-167 cc | 0 | 16 | 1 |
| 1094 | 2001 | NG | ST-884 | ST-167 cc | 0 | 23 | 1 |
| 1095 | 2001 | NG | ST-1636 | ST-167 cc | 0 | 17 | 1 |
| 1096 | 2001 | NG | ST-198 | ST-198 cc | 0 | 18 | 0 |
| 1097 | 2001 | NG | ST-198 | ST-198 cc | 0 | 16 | 0 |
| 1098 | 2001 | NG | ST-198 | ST-198 cc | 0 | 17 | 0 |
| 1099 | 2001 | NG | ST-198 | ST-198 cc | 0 | 16 | 0 |
| 1100 | 2001 | NG | ST-198 | ST-198 cc | 0 | 17 | 0 |
| 1101 | 2001 | NG | ST-198 | ST-198 cc | 0 | 17 | 0 |
| 1102 | 2001 | NG | ST-198 | ST-198 cc | 0 | 16 | 0 |
| 1103 | 2001 | NG | ST-198 | ST-198 cc | 0 | 17 | 0 |
| 1104 | 2001 | NG | ST-198 | ST-198 cc | 0 | 17 | 0 |
| 1105 | 2001 | NG | ST-823 | ST-198 cc | 0 | 17 | 0 |
| 1106 | 2001 | NG | ST-198 | ST-198 cc | 0 | 18 | 1 |
| 1107 | 2001 | B | ST-213 | ST-213 cc | 0 | 16 | 0 |
| 1108 | 2001 | B | ST-213 | ST-213 cc | 0 | 17 | 0 |
| 1109 | 2001 | B | ST-213 | ST-213 cc | 0 | 17 | 0 |
| 1110 | 2001 | B | ST-213 | ST-213 cc | 0 | 16 | 0 |
| 1111 | 2001 | B | ST-213 | ST-213 cc | 0 | 17 | 0 |
| 1112 | 2001 | B | ST-213 | ST-213 cc | 0 | 16 | 0 |
| 1113 | 2001 | B | ST-213 | ST-213 cc | 0 | 18 | 0 |
| 1114 | 2001 | B | ST-213 | ST-213 cc | 0 | 16 | 0 |
| 1115 | 2001 | B | ST-213 | ST-213 cc | 0 | 16 | 0 |
| 1116 | 2001 | B | ST-213 | ST-213 cc | 0 | 19 | 0 |
| 1117 | 2001 | B | ST-213 | ST-213 cc | 0 | 16 | 0 |
| 1118 | 2001 | B | ST-213 | ST-213 cc | 0 | 18 | 0 |
| 1119 | 2001 | B | ST-213 | ST-213 cc | 0 | 16 | 0 |
| 1120 | 2001 | NG | ST-213 | ST-213 cc | 0 | 16 | 0 |
| 1121 | 2001 | NG | ST-213 | ST-213 cc | 0 | 16 | 0 |
| 1122 | 2001 | NG | ST-213 | ST-213 cc | 0 | 17 | 0 |
| 1123 | 2001 | NG | ST-213 | ST-213 cc | 0 | 18 | 0 |
| 1124 | 2001 | NG | ST-213 | ST-213 cc | 0 | 17 | 0 |
| 1125 | 2001 | NG | ST-213 | ST-213 cc | 0 | 17 | 0 |
| 1126 | 2001 | B | ST-2388 | ST-213 cc | 0 | 17 | 0 |
| 1127 | 2001 | B | ST-2391 | ST-213 cc | 0 | 17 | 0 |
| 1128 | 2001 | NG | ST-2391 | ST-213 cc | 0 | 17 | 0 |
| 1129 | 2001 | B | ST-3115 | ST-213 cc | 0 | 17 | 0 |
| 1130 | 2001 | B | ST-213 | ST-213 cc | 0 | 18 | 1 |
| 1131 | 2001 | B | ST-213 | ST-213 cc | 0 | 16 | 0 |
| 1132 | 2001 | NG | ST-213 | ST-213 cc | 0 | 17 | 1 |
| 1133 | 2001 | NG | ST-1943 | ST-213 cc | 0 | 16 | 0 |
| 1134 | 2001 | B | ST-2389 | ST-213 cc | 0 | 17 | 0 |
| 1135 | 2001 | 29E | ST-254 | ST-254 cc | 0 | 16 | 0 |
| 1136 | 2001 | 29E | ST-254 | ST-254 cc | 0 | 18 | 0 |
| 1137 | 2001 | NG | ST-2629 | ST-254 cc | 0 | 17 | 0 |
| 1138 | 2001 | 29E | ST-3811 | ST-254 cc | 0 | 17 | 0 |
| 1139 | 2001 | C | ST-2629 | ST-254 cc | 0 | 16 | 1 |
| 1140 | 2001 | B | ST-275 | ST-269 cc | 0 | 17 | 0 |
| 1141 | 2001 | B | ST-269 | ST-269 cc | 0 | 16 | 1 |
| 1142 | 2001 | B | ST-269 | ST-269 cc | 0 | 17 | 1 |
| 1143 | 2001 | NG | ST-269 | ST-269 cc | 0 | 17 | 1 |
| 1144 | 2001 | NG | ST-269 | ST-269 cc | 0 | 17 | 1 |
| 1145 | 2001 | NG | ST-269 | ST-269 cc | 0 | 16 | 1 |
| 1146 | 2001 | NG | ST-269 | ST-269 cc | 0 | 17 | 1 |
| 1147 | 2001 | B | ST-275 | ST-269 cc | 0 | 16 | 1 |
| 1148 | 2001 | B | ST-275 | ST-269 cc | 0 | 16 | 1 |
| 1149 | 2001 | B | ST-275 | ST-269 cc | 0 | 16 | 1 |
| 1150 | 2001 | B | ST-275 | ST-269 cc | 0 | 17 | 1 |
| 1151 | 2001 | NG | ST-275 | ST-269 cc | 0 | 17 | 1 |
| 1152 | 2001 | NG | ST-275 | ST-269 cc | 0 | 16 | 1 |
| 1153 | 2001 | NG | ST-275 | ST-269 cc | 0 | 17 | 1 |
| 1154 | 2001 | NG | ST-275 | ST-269 cc | 0 | 16 | 1 |
| 1155 | 2001 | C | ST-467 | ST-269 cc | 0 | 16 | 1 |
| 1156 | 2001 | NG | ST-467 | ST-269 cc | 0 | 18 | 1 |
| 1157 | 2001 | NG | ST-467 | ST-269 cc | 0 | 17 | 1 |
| 1158 | 2001 | NG | ST-467 | ST-269 cc | 0 | 18 | 1 |
| 1159 | 2001 | B | ST-492 | ST-269 cc | 0 | 17 | 1 |
| 1160 | 2001 | NG | ST-492 | ST-269 cc | 0 | 17 | 1 |
| 1161 | 2001 | B | ST-1163 | ST-269 cc | 0 | 17 | 1 |
| 1162 | 2001 | B | ST-2091 | ST-269 cc | 0 | 17 | 1 |
| 1163 | 2001 | NG | ST-2368 | ST-269 cc | 0 | 17 | 1 |
| 1164 | 2001 | NG | ST-2369 | ST-269 cc | 0 | 17 | 1 |
| 1165 | 2001 | NG | ST-2369 | ST-269 cc | 0 | 17 | 1 |
| 1166 | 2001 | NG | ST-3082 | ST-269 cc | 0 | 16 | 1 |
| 1167 | 2001 | B | ST-3216 | ST-364 cc | 0 | 17 | 1 |
| 1168 | 2001 | B | ST-461 | ST-461 cc | 0 | 17 | 0 |
| 1169 | 2001 | NG | ST-750 | ST-750 cc | 0 | 17 | 1 |
| 1170 | 2001 | X | ST-750 | ST-750 cc | 0 | 16 | 1 |
| 1171 | 2001 | X | ST-750 | ST-750 cc | 0 | 17 | 1 |
| 1172 | 2001 | NG | ST-1339 | ST-750 cc | 0 | 16 | 1 |
| 1173 | 2001 | X | ST-1339 | ST-750 cc | 0 | 17 | 1 |
| 1174 | 2001 | X | ST-1339 | ST-750 cc | 0 | 17 | 1 |
| 1175 | 2001 | NG | ST-1157 | ST-1157 cc | 0 | 16 | 0 |
| 1176 | 2001 | NG | ST-3153 | ST-1157 cc | 0 | 16 | 0 |
| 1177 | 2001 | 29E | ST-1157 | ST-1157 cc | 0 | 17 | 1 |
| 1178 | 2001 | B | ST-1157 | ST-1157 cc | 0 | 18 | 1 |
| 1179 | 2001 | NG | ST-1157 | ST-1157 cc | 0 | 16 | 1 |
| 1180 | 2001 | NG | ST-1157 | ST-1157 cc | 0 | 18 | 1 |
| 1181 | 2001 | NG | ST-1157 | ST-1157 cc | 0 | 17 | 1 |
| 1182 | 2001 | NG | ST-1157 | ST-1157 cc | 0 | 16 | 1 |
| 1183 | 2001 | NG | ST-1157 | ST-1157 cc | 0 | 18 | 1 |
| 1184 | 2001 | NG | ST-1157 | ST-1157 cc | 0 | 17 | 1 |
| 1185 | 2001 | NG | ST-1157 | ST-1157 cc | 0 | 17 | 1 |
| 1186 | 2001 | NG | ST-1157 | ST-1157 cc | 0 | 17 | 1 |
| 1187 | 2001 | NG | ST-1157 | ST-1157 cc | 0 | 15 | 1 |
| 1188 | 2001 | NG | ST-1157 | ST-1157 cc | 0 | 16 | 1 |
| 1189 | 2001 | NG | ST-1157 | ST-1157 cc | 0 | 17 | 1 |
| 1190 | 2001 | NG | ST-1157 | ST-1157 cc | 0 | 16 | 1 |
| 1191 | 2001 | NG | ST-1157 | ST-1157 cc | 0 | 17 | 1 |
| 1192 | 2001 | NG | ST-1157 | ST-1157 cc | 0 | 16 | 1 |
| 1193 | 2001 | NG | ST-1157 | ST-1157 cc | 0 | 18 | 1 |
| 1194 | 2001 | NG | ST-1157 | ST-1157 cc | 0 | 16 | 1 |
| 1195 | 2001 | NG | ST-1157 | ST-1157 cc | 0 | 16 | 1 |
| 1196 | 2001 | NG | ST-1157 | ST-1157 cc | 0 | 16 | 1 |
| 1197 | 2001 | NG | ST-1157 | ST-1157 cc | 0 | 16 | 1 |
| 1198 | 2001 | NG | ST-1157 | ST-1157 cc | 0 | 16 | 1 |
| 1199 | 2001 | NG | ST-1157 | ST-1157 cc | 0 | 16 | 1 |
| 1200 | 2001 | NG | ST-1157 | ST-1157 cc | 0 | 16 | 1 |
| 1201 | 2001 | NG | ST-1157 | ST-1157 cc | 0 | 16 | 1 |
| 1202 | 2001 | NG | ST-1157 | ST-1157 cc | 0 | 17 | 1 |
| 1203 | 2001 | NG | ST-1157 | ST-1157 cc | 0 | 16 | 1 |
| 1204 | 2001 | NG | ST-1157 | ST-1157 cc | 0 | 16 | 1 |
| 1205 | 2001 | NG | ST-1421 | ST-1157 cc | 0 | 17 | 1 |
| 1206 | 2001 | NG | ST-3150 | ST-1157 cc | 0 | 17 | 1 |
| 1207 | 2001 | NG | ST-3150 | ST-1157 cc | 0 | 16 | 1 |
| 1208 | 2001 | B | ST-3152 | ST-1157 cc | 0 | 18 | 1 |
| 1209 | 2001 | B | ST-286 | ST-nd cc | 0 | 17 | 0 |
| 1210 | 2001 | NG | ST-963 | ST-nd cc | 0 | 16 | 0 |
| 1211 | 2001 | NG | ST-963 | ST-nd cc | 0 | 16 | 0 |
| 1212 | 2001 | NG | ST-963 | ST-nd cc | 0 | 16 | 0 |
| 1213 | 2001 | NG | ST-963 | ST-nd cc | 0 | 16 | 0 |
| 1214 | 2001 | NG | ST-963 | ST-nd cc | 0 | 16 | 0 |
| 1215 | 2001 | NG | ST-963 | ST-nd cc | 0 | 17 | 0 |
| 1216 | 2001 | NG | ST-963 | ST-nd cc | 0 | 16 | 0 |
| 1217 | 2001 | NG | ST-1117 | ST-nd cc | 0 | 16 | 0 |
| 1218 | 2001 | NG | ST-1117 | ST-nd cc | 0 | 16 | 0 |
| 1219 | 2001 | NG | ST-1117 | ST-nd cc | 0 | 16 | 0 |
| 1220 | 2001 | NG | ST-1117 | ST-nd cc | 0 | 17 | 0 |
| 1221 | 2001 | NG | ST-1117 | ST-nd cc | 0 | 16 | 0 |
| 1222 | 2001 | NG | ST-1117 | ST-nd cc | 0 | 16 | 0 |
| 1223 | 2001 | NG | ST-1117 | ST-nd cc | 0 | 16 | 0 |
| 1224 | 2001 | NG | ST-1117 | ST-nd cc | 0 | 16 | 0 |
| 1225 | 2001 | NG | ST-1117 | ST-nd cc | 0 | 16 | 0 |
| 1226 | 2001 | NG | ST-1117 | ST-nd cc | 0 | 16 | 0 |
| 1227 | 2001 | NG | ST-1117 | ST-nd cc | 0 | 15 | 0 |
| 1228 | 2001 | NG | ST-1117 | ST-nd cc | 0 | 18 | 0 |
| 1229 | 2001 | NG | ST-1136 | ST-nd cc | 0 | 16 | 0 |
| 1230 | 2001 | NG | ST-1136 | ST-nd cc | 0 | 17 | 0 |
| 1231 | 2001 | NG | ST-1431 | ST-nd cc | 0 | 16 | 0 |
| 1232 | 2001 | NG | ST-1630 | ST-nd cc | 0 | 17 | 0 |
| 1233 | 2001 | B | ST-1653 | ST-nd cc | 0 | 18 | 0 |
| 1234 | 2001 | NG | ST-1975 | ST-nd cc | 0 | 17 | 1 |
| 1235 | 2001 | NG | ST-2183 | ST-nd cc | 0 | 17 | 0 |
| 1236 | 2001 | NG | ST-2461 | ST-nd cc | 0 | 18 | 0 |
| 1237 | 2001 | NG | ST-3058 | ST-nd cc | 0 | 16 | 1 |
| 1238 | 2001 | 29E | ST-3207 | ST-nd cc | 0 | 16 | 0 |
| 1239 | 2001 | NG | ST-3207 | ST-nd cc | 0 | 17 | 0 |
| 1240 | 2001 | B | ST-3213 | ST-nd cc | 0 | 17 | 0 |
| 1241 | 2001 | 29E | ST-3238 | ST-nd cc | 0 | 16 | 0 |
| 1242 | 2001 | NG | ST-3258 | ST-nd cc | 0 | 17 | 0 |
| 1243 | 2001 | NG | ST-3275 | ST-nd cc | 0 | 17 | 0 |
| 1244 | 2001 | NG | ST-4067 | ST-nd cc | 0 | 16 | 0 |
| 1245 | 2001 | NG | ST-178 | ST-nd cc | 0 | 16 | 1 |
| 1246 | 2001 | NG | ST-178 | ST-nd cc | 0 | 16 | 1 |
| 1247 | 2001 | NG | ST-178 | ST-nd cc | 0 | 16 | 1 |
| 1248 | 2001 | NG | ST-178 | ST-nd cc | 0 | 18 | 1 |
| 1249 | 2001 | NG | ST-178 | ST-nd cc | 0 | 18 | 1 |
| 1250 | 2001 | B | ST-282 | ST-nd cc | 0 | 17 | 1 |
| 1251 | 2001 | NG | ST-282 | ST-nd cc | 0 | 16 | 1 |
| 1252 | 2001 | NG | ST-963 | ST-nd cc | 0 | 16 | 0 |
| 1253 | 2001 | NG | ST-1052 | ST-nd cc | 0 | 18 | 1 |
| 1254 | 2001 | NG | ST-1117 | ST-nd cc | 0 | 17 | 1 |
| 1255 | 2001 | W135 | ST-1117 | ST-nd cc | 0 | 17 | 1 |
| 1256 | 2001 | NG | ST-1136 | ST-nd cc | 0 | 16 | 0 |
| 1257 | 2001 | NG | ST-1136 | ST-nd cc | 0 | 17 | 0 |
| 1258 | 2001 | B | ST-1167 | ST-nd cc | 0 | 17 | 1 |
| 1259 | 2001 | NG | ST-1167 | ST-nd cc | 0 | 16 | 1 |
| 1260 | 2001 | NG | ST-1167 | ST-nd cc | 0 | 16 | 1 |
| 1261 | 2001 | NG | ST-1167 | ST-nd cc | 0 | 16 | 1 |
| 1262 | 2001 | B | ST-1879 | ST-nd cc | 0 | 17 | 1 |
| 1263 | 2001 | NG | ST-1879 | ST-nd cc | 0 | 17 | 1 |
| 1264 | 2001 | B | ST-1975 | ST-nd cc | 0 | 16 | 1 |
| 1265 | 2001 | B | ST-2086 | ST-nd cc | 0 | 17 | 1 |
| 1266 | 2001 | NG | ST-2086 | ST-nd cc | 0 | 16 | 1 |
| 1267 | 2001 | NG | ST-2196 | ST-nd cc | 0 | 17 | 1 |
| 1268 | 2001 | B | ST-2378 | ST-nd cc | 0 | 18 | 1 |
| 1269 | 2001 | NG | ST-2393 | ST-nd cc | 0 | 17 | 1 |
| 1270 | 2001 | NG | ST-2393 | ST-nd cc | 0 | 16 | 1 |
| 1271 | 2001 | NG | ST-2394 | ST-nd cc | 0 | 16 | 0 |
| 1272 | 2001 | NG | ST-2395 | ST-nd cc | 0 | 17 | 1 |
| 1273 | 2001 | NG | ST-2457 | ST-nd cc | 0 | 17 | 1 |
| 1274 | 2001 | B | ST-3067 | ST-nd cc | 0 | 17 | 1 |
| 1275 | 2001 | B | ST-3089 | ST-nd cc | 0 | 16 | 1 |
| 1276 | 2001 | NG | ST-3110 | ST-nd cc | 0 | 21 | 1 |
| 1277 | 2001 | NG | ST-3128 | ST-nd cc | 0 | 16 | 1 |
| 1278 | 2001 | NG | ST-3128 | ST-nd cc | 0 | 17 | 1 |
| 1279 | 2001 | NG | ST-3130 | ST-nd cc | 0 | 17 | 1 |
| 1280 | 2001 | NG | ST-3131 | ST-nd cc | 0 | 16 | 1 |
| 1281 | 2001 | B | ST-3241 | ST-nd cc | 0 | 18 | 1 |
| 1282 | 2001 | NG | ST-3256 | ST-nd cc | 0 | 17 | 1 |
| 1283 | 2001 | NG | ST-3263 | ST-nd cc | 0 | 17 | 1 |
| 1284 | 2001 | NG | ST-3266 | ST-nd cc | 0 | 17 | 0 |
| 1285 | 2001 | NG | ST-3276 | ST-nd cc | 0 | 17 | 1 |
| 1286 | 2001 | NG | ST-4424 | ST-nd cc | 0 | 17 | 1 |
| 1287 | 2001 | NG | ST-4543 | ST-nd cc | 0 | 16 | 1 |
| 1288 | 2001 | NG | ST-4543 | ST-nd cc | 0 | 17 | 1 |
