# Supplementary material for: Association of a Bacteriophage with Meningococcal Disease in Young Adults
Source: PLoS One. 2008 Dec 9;3(12):e3885. doi: 10.1371/journal.pone.0003885 (PMC2587699; doi:10.1371/journal.pone.0003885)
Supplement: Table S2 — Distribution of the MDA island in meningococci isolated from patients and carriers before (1999) and after (2001) the vaccination campaign (0.13 MB DOC) [file pone.0003885.s002.doc]

| **Table S2** | | | | | | |
| --- | --- | --- | --- | --- | --- | --- |
| Distribution of the MDA island in meningococci isolated from patients and carriers before (1999) and after (2001) the vaccination campaign. | | | | | | |
|  | | | | | | |
|  |  | Carried | |  | Case | |
| clonal complex |  | Pre- vaccination | Post-vaccination |  | Pre- vaccination | Post-vaccination |
| ST-5 (mda -) |  | 0 | 0 |  | 1 (0,5%) ^a^ | 0 |
| ST-5 (MDA+) |  | 0 | 0 |  | 0 | 0 |
| ST-8 (mda -) |  | 0 | 1 (0,2%) |  | 1 (0,5%) | 0 |
| ST-8 (MDA+) |  | 1 (0,2%) | 1 (0,2%) |  | 9 (4,4%) | 2 (1,3%) |
| ST-11 (mda -) |  | 0 | 0 |  | 0 | 2 (1,3%) |
| ST-11 (MDA+) |  | 10 (2%) | 1 (0,2%) |  | 64 (31,5%) | 32 (20,8%) |
| ST-18 (mda -) |  | 0 | 0 |  | 3 (1,5%) | 0 |
| ST-18 (MDA+) |  | 0 | 0 |  | 1 (0,5%) | 1 (0,6%) |
| ST-22 (mda -) |  | 7 (1,4%) | 48 (11,1%) |  | 8 (3,9%) | 2 (1,3%) |
| ST-22 (MDA+) |  | 48 (9,6%) | 4 (0,9%) |  | 1 (0,5%) | 0 |
| ST-23 (mda -) |  | 2 (0,4%) | 8 (1,9%) |  | 3 (1,5%) | 0 |
| ST-23 (MDA+) |  | 32 (6,4%) | 9 (2,1%) |  | 0 | 0 |
| ST-32 (mda -) |  | 0 | 0 |  | 0 | 1 (0,6%) |
| ST-32 (MDA+) |  | 6 (1,2%) | 6 (1,4%) |  | 7 (3,4%) | 10 (6,5%) |
| ST-35 (mda -) |  | 2 (0,4%) | 1 (0,2%) |  | 0 | 0 |
| ST-35 (MDA+) |  | 14 (2,8%) | 15 (3,5%) |  | 1 (0,5%) | 2 (1,3%) |
| ST-41/44 (mda -) |  | 3 (0,6%) | 0 |  | 5 (2,5%) | 0 |
| ST-41/44 (MDA+) |  | 58 (11,6%) | 47 (10,9%) |  | 52 (25,6%) | 41 (26,6%) |
| ST-53 (mda -) |  | 38 (7,6%) | 24 (5,6%) |  | 0 | 0 |
| ST-53 (MDA+) |  | 0 | 11 (2,6%) |  | 0 | 0 |
| ST-60 (mda -) |  | 34 (6,8%) | 2 (0,5%) |  | 7 (3,4%) | 2 (1,3%) |
| ST-60 (MDA+) |  | 6 (1,2%) | 34 (7,9%) |  | 1 (0,5%) | 0 |
| ST-92 (mda -) |  | 0 | 0 |  | 0 | 0 |
| ST-92 (MDA+) |  | 0 | 1 (0,2%) |  | 0 | 0 |
| ST-103 (mda -) |  | 7 (1,4%) | 3 (0,7%) |  | 0 | 1 (0,6%) |
| ST-103 (MDA+) |  | 3 (0,6%) | 7 (1,6%) |  | 0 | 0 |
| ST-162 (mda -) |  | 0 | 0 |  | 0 | 0 |
| ST-162 (MDA+) |  | 8 (1,6%) | 2 (0,5%) |  | 1 (0,5%) | 0 |
| ST-167 (mda -) |  | 21 (4,2%) | 2 (0,5%) |  | 1 (0,5%) | 0 |
| ST-167 (MDA+) |  | 3 (0,6%) | 11 (2,6%) |  | 1 (0,5%) | 1 (0,6%) |
| ST-198 (mda -) |  | 17 (3,4%) | 10 (2,3%) |  | 0 | 0 |
| ST-198 (MDA+) |  | 1 (0,2%) | 1 (0,2%) |  | 0 | 0 |
| ST-213 (mda -) |  | 22 (4,4%) | 26 (6%) |  | 1 (0,5%) | 2 (1,3%) |
| ST-213 (MDA+) |  | 1 (0,2%) | 2 (0,5%) |  | 1 (0,5%) | 1 (0,6%) |
| ST-254 (mda -) |  | 6 (1,2%) | 4 (0,9%) |  | 0 | 0 |
| ST-254 (MDA+) |  | 1 (0,2%) | 1 (0,2%) |  | 0 | 0 |
| ST-269 (mda -) |  | 1 (0,2%) | 1 (0,2%) |  | 3 (1,5%) | 1 (0,6%) |
| ST-269 (MDA+) |  | 30 (6%) | 26 (6%) |  | 25 (12,3%) | 31 (20,1%) |
| ST-334 (mda -) |  | 1 (0,2%) | 0 |  | 0 | 0 |
| ST-334 (MDA+) |  | 0 | 0 |  | 0 | 0 |
| ST-364 (mda -) |  | 1 (0,2%) | 0 |  | 0 | 0 |
| ST-364 (MDA+) |  | 0 | 1 (0,2%) |  | 1 (0,5%) | 1 (0,6%) |
| ST-461 (mda -) |  | 0 | 1 (0,2%) |  | 1 (0,5%) | 1 (0,6%) |
| ST-461 (MDA+) |  | 0 | 0 |  | 0 | 0 |
| ST-750 (mda -) |  | 0 | 0 |  | 0 | 0 |
| ST-750 (MDA+) |  | 5 (1%) | 6 (1,4%) |  | 0 | 0 |
| ST-865 (mda -) |  | 3 (0,6%) | 0 |  | 0 | 0 |
| ST-865 (MDA+) |  | 0 | 0 |  | 0 | 0 |
| ST-1157 (mda -) |  | 3 (0,6%) | 2 (0,5%) |  | 0 | 1 (0,6%) |
| ST-1157 (MDA+) |  | 26 (5,2%) | 32 (7,4%) |  | 1 (0,5%) | 0 |
| nd^b^ (mda -) |  | 40 (8%) | 39 (9%) |  | 2 (1%) | 5 (3,2%) |
| nd (MDA+) |  | 39 (7,8%) | 41 (9,5%) |  | 1 (0,5%) | 14 (9,1%) |

a) The number of isolates is followed by the corresponding percentage with relation to the group defined at the head of the column.

b) nd: A clonal complex has not been defined for organisms of certain sequence types. In this study these sequence types tended to be represented by only a few isolates. Hence isolates belonging to these sequence types are grouped together in the analysis.
